# Comparative Study of Data Matrix Codes Localization and Recognition Methods

**DOI:** 10.3390/jimaging7090163

**Published:** 2021-08-27

**Authors:** Ladislav Karrach, Elena Pivarčiová

**Affiliations:** Department of Manufacturing and Automation Technology, Faculty of Technology, Technical University in Zvolen, Masarykova 24, 96001 Zvolen, Slovakia; karrach@zoznam.sk

**Keywords:** Data Matrix code recognition, edge detection, adaptive thresholding, finder pattern, timing pattern, perspective distortion

## Abstract

We provide a comprehensive and in-depth overview of the various approaches applicable to the recognition of Data Matrix codes in arbitrary images. All presented methods use the typical “L” shaped Finder Pattern to locate the Data Matrix code in the image. Well-known image processing techniques such as edge detection, adaptive thresholding, or connected component labeling are used to identify the Finder Pattern. The recognition rate of the compared methods was tested on a set of images with Data Matrix codes, which is published together with the article. The experimental results show that methods based on adaptive thresholding achieved a better recognition rate than methods based on edge detection.

## 1. Introduction

The popularity and use of two-dimensional (2D) matrix codes are growing and they are replacing traditional linear 1D barcodes in many areas of life. The most important advantages of 2D codes are the data capacity (the ability to store more data in a smaller area) and the error correction ability (by utilizing Reed-Solomon algorithm). A 2D code attached to the product can contain detailed information about the product, manufacturer, recipient, customer, and therefore find uses in production, inventory, distribution, sales, and repair processes.

Two-dimensional matrix codes are built of black and white modules (also called cells), usually arranged in a square pattern. One module represents the smallest building block and, in the data area, the dark module usually encodes binary 1 and the light module binary 0. As more data is encoded in a 2D code, the number of modules (rows and columns) increases.

**Figure 1:**
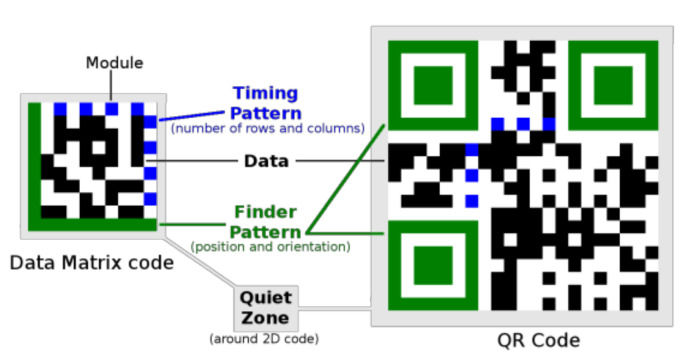
Structure of Data Matrix code compared to QR Code.

Each type of 2D code has its characteristic fixed parts, which are used to determine its position and orientation (Finder Pattern) and to determine its dimensions (Timing Pattern). Variable parts encode data (Figure 1).

Data Matrix and QR Codes are the most common types of 2D codes (Table 1). Data Matrix codes are usually used to mark small items such as electronic components because they need less space to encode the same amount of data.

### Related Work

Recently, relatively few articles have been published dealing with the recognition of Data Matrix codes in images (most authors focus on recognizing QR codes). Qiang Huang et al. in [1] presented the Finder Pattern detection method mainly based on line segment detection [2] and combination. Donghong et al. [3] proposed an algorithm based on the Radon transform and Chenguang et al. [4] proposed the algorithm based on the Hough transform (both of these algorithms are time-consuming). Liu et al. [5] combined connected region selection, based on region feature analysis, and positioning using the line Snake algorithm. Cho et al. [6] utilized the Selective Search method to detect candidate regions, followed by LBP (Local Binary Patterns) and HOG (Histogram of Oriented Gradients) feature extraction and SVM (Support Vector Machine) classification to determine 2D code regions. Sörös [7] suggested a 2D code localization algorithm, which combines the search for areas with a high concentration of edge structures as well as for areas with a high concentration of corner structures.

## 2. The Data Matrix Code Localization Methods

**Figure 2:**
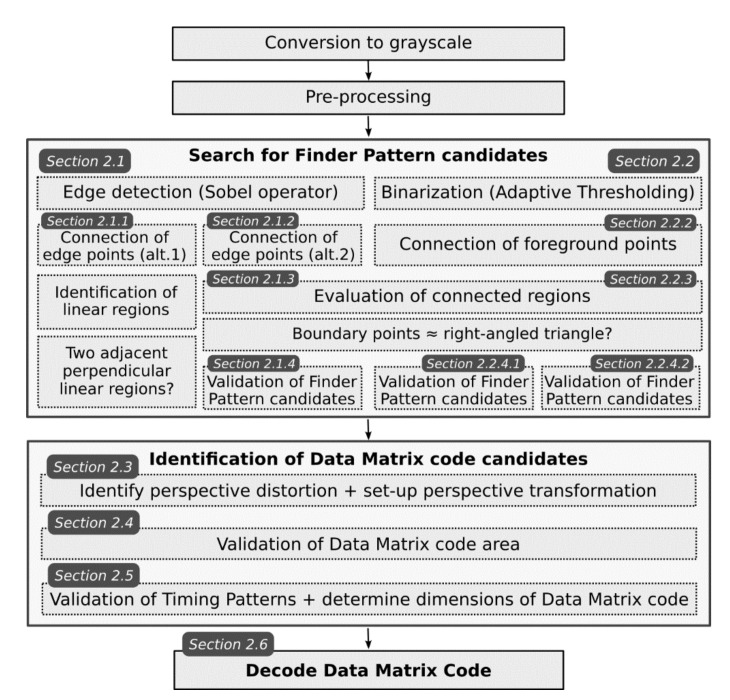
The flow chart of the proposed algorithms.

In the following sections, we will give an overview of several methods for localization Data Matrix codes in images which all utilize a typical “L” shaped Finder Pattern. The individual steps of the Data Matrix code localization algorithms are schematically shown in Figure 2 (some steps are common to different algorithms/methods).

Finder Pattern localization is based on the assumption that the Finder Pattern area is darker than its surroundings and therefore can be segmented based on the gray intensity. Two basic image processing techniques are utilized:

- Edge detection (see Section 2.1)
- Adaptive thresholding (see Section 2.2)

The procedures described in Section 2.3, Section 2.4, Section 2.5 and Section 2.6 are common and follow up the procedures described in Section 2.1 and Section 2.2.

### 2.1. Edge Detection Methods (Method Group 1)

An input grayscale image is convolved using a 3 × 3 Sobel operator [8]. The Sobel operator approximates the first derivates of the image function and uses two kernels to approximate horizontal *G_x_* and vertical *G_y_* derivate at each point in the input image *I* Equation (1).

(1) $$G_{x}=\left[\begin{array}{ccc} -1 & 0 & +1\\ -2 & 0 & +2\\ -1 & 0 & +1 \end{array}\right]*I,\;G_{y}=\left[\begin{array}{ccc} -1 & -2 & -1\\ 0 & 0 & 0\\ +1 & +2 & +1 \end{array}\right]*I$$

where *G_x_*, *G_y_* are images approximating horizontal and vertical derivates, respectively, and *I* is an input grayscale image.

**Figure 3:**
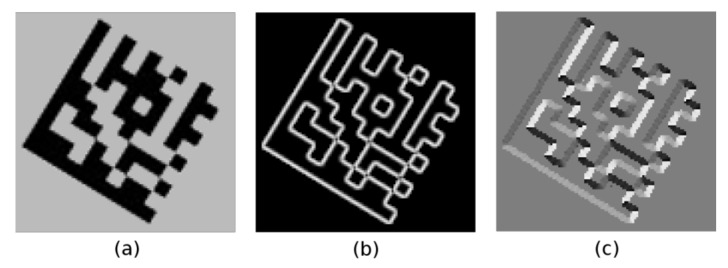
(**a**) grayscale image; (**b**) gradient magnitude image; (**c**) gradient direction image.

Using these gradient images, the gradient magnitude image *M* (Figure 3b) and the gradient direction image *A* (Figure 3c) are computed Equation (2).

(2) $$M=\sqrt{G_{x}^{2}+G_{y}^{2}},\;A=\arctan\left(\frac{G_{y}}{G_{x}}\right)$$

A gradient direction is perpendicular to a direction of the edge.

#### 2.1.1. Connecting of Edge Points into Continuous Regions (Alternative 1)

A modified 2-pass connected component labeling algorithm [9] is applied to the gradient images. Edge points that meet all of the following conditions are joined into continuous regions:

- Gradient magnitude of edge point (in the gradient magnitude image *M*) must be above fixed limit 90 (weak edges caused by noise are ignored);
- Gradient direction of edge point (in the gradient direction image *A*) must not differ by more than 22 degrees from the average angle of the region (i.e., the average of gradient directions of edge points that have been joined into the given region so far);
- The intensity of at least one neighboring point in the grayscale image *I* is under fixed value 110 (Data Matrix code must be an object dark enough in an image).

A region descriptor is maintained for each continuous region (BLOB). As the individual edge points (at coordinates *x*, *y*) are added to the given continuous region, the region descriptor is updated as follows:

- *Area* ← *Area* + 1 (*M*_00_ ← *M*_00_ + 1);
- An outer bounding box: *Top*, *Right, Bottom* and *Left;*
- A sum of gradient directions (angles): *Angles* ← *Angles* + *A* (*x*, *y*);
- Region moments: *M*_10_ ← *M*_10_ + *x*, *M*_01_ ← *M*_01_ + *y*, *M*_11_ ← *M*_11_ + *x* × *y*, *M*_20_ ← *M*_20_ + *x* × *x*, *M*_02_ ← *M*_02_ + *y* × *y.*

**Figure 4:**
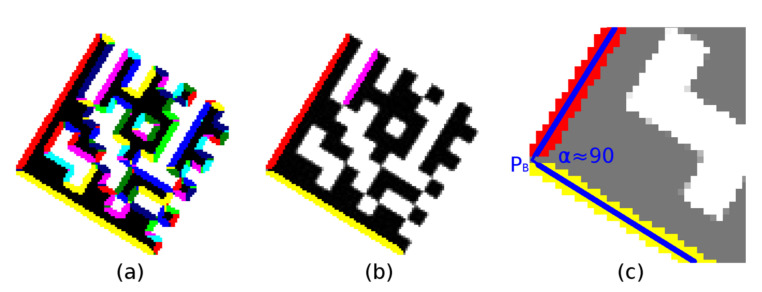
(**a**) colored continuous regions; (**b**,**c**) significant linear regions.

After all the regions are labeled (Figure 4a), the region moments are used to compute the region centroid *C* and the region orientation *Θ* (Equation (3); region orientation is the angle between the *x*-axis and the major axis of the region).

(3) $$C=\left(\frac{M_{10}}{M_{00}},\frac{M_{01}}{M_{00}}\right)=\left(C_{x},C_{y}\right),\;\;\Theta =\frac{1}{2}\arctan\left(\frac{2\mu_{11}}{\mu_{20}-\mu_{02}}\right)=\frac{1}{2}\arctan\left(\frac{2\left(M_{11}-C_{x}M_{01}\right)}{\left(M_{20}-C_{x}M_{10}\right)-\left(M_{02}-C_{y}M_{01}\right)}\right)$$

**Figure 5:**
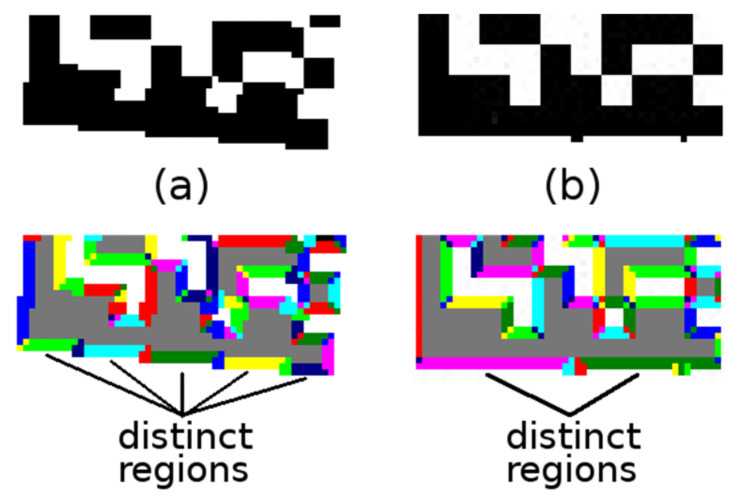
(**a**) “sharp stairs”; (**b**) “bumpy edges”.

The modification of 2-pass connected component labeling algorithm consists of:

- Adding an intermediate step, where also nearby (their distance is at most 2 points) distinct regions of a similar direction (their difference in angles is at most 7.5 degrees) are marked as equivalent (Figure 5a; this situation can occur with synthetic Data Matrix codes, where there are “sharp stairs”);
- Adding a finalization step, where distinct regions which have end-points that are at most three points apart and the angle *Θ* (Equation (3)) of regions differs by less than five degrees and the major axis of one region is not more than 1.5 points away from the centroid *C* (Equation (3)) of the other region are joined (Figure 5b; this situation can occur when there are “bumpy edges”).

Evaluation of connected regions—filtering out non-linear regions.

Region descriptors are evaluated and small regions with an area of less than 40 are filtered out first. For the remaining regions, it is verified whether it is possible to put a line passing through the region’s centroid *C* at an angle *Θ* (Equation (3)) and having at least 90% of the points in common with the region and the length of this line segment is at least 20 points (Figure 4b). The common points of the line and the region form the axis of the region with the two end-points.

Searching for Finder Pattern candidates—two adjacent perpendicular linear regions.

Region descriptors are scanned to find pairs of regions perpendicular to each other (the difference between the right angle and the differences of the region angles *Θ* is less than five degrees; if perspective distorted Data Matrix codes must be considered, the tolerance must be increased accordingly), the distance of the regions end-points is less than five points and their length does not differ by more than 10% (Figure 4c). The intersection of the axes of the regions is the vertex (*P*_B_) of the Finder Pattern.

Processing continues in Section 2.3.

#### 2.1.2. Connecting of Edge Points into Continuous Regions (Alternative 2)

**Figure 6:**
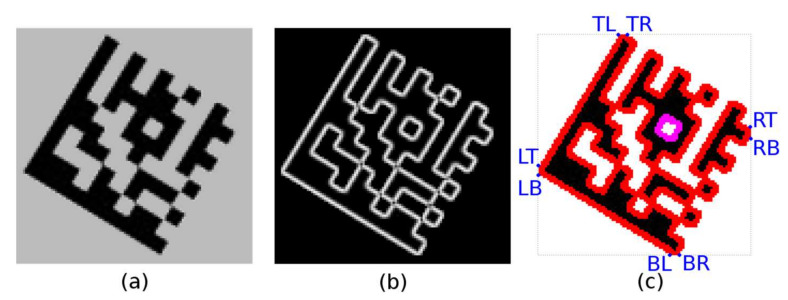
(**a**) grayscale image; (**b**) edge image (magnitudes); (**c**) colored continuous edge regions.

The connected component labeling algorithm [9] is applied to the edge (magnitude) image *M* (Figure 6b) obtained in the edge detection step (2.1; in contrast to the previous Alternative 1, the orientation of the edges—gradient direction image *A*—is not taken into account). A region descriptor is maintained for each continuous region (BLOB). As the individual edge points are added to the given continuous region, it is updated as follows:

- *Area* ← *Area* + 1;
- (*x*, *y*) coordinates of the added point are used to update 8 boundary points: *Top*-*Left*, *Top*-*Right*, *Right*-*Top*, *Right*-*Bottom*, *Bottom*-*Right*, *Bottom*-*Left*, *Left*-*Bottom*, *Left*-*Top.*

Only the edge points which have a magnitude that is above a specified threshold are considered. The threshold was experimentally set to 70 to ensure that weak edges are ignored.

#### 2.1.3. Evaluation of Finder Pattern Candidates

Region descriptors computed during the connected component labeling phase are used to filter out regions that cannot represent the outer edges of the Finder Pattern. The candidate region must have a minimum area and such three boundary points (out of 8 boundary points; Figure 6c) which form an isosceles right-angled triangle. This procedure is described in detail in Section 2.2.3.

#### 2.1.4. Validating of Finder Pattern Candidates and Aligning to Finder Pattern

**Figure 7:**
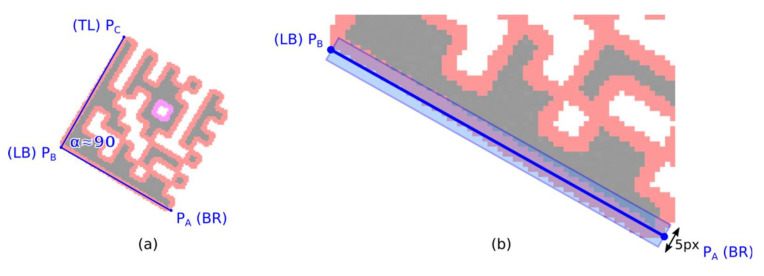
(**a**) three vertices (P_A_-P_B_-P_C_) of an isosceles right-angled triangle; (**b**) optimize position of the line segments P_A_-P_B_ (P_B_-P_C_) using region moments.

A Finder Pattern candidate region is described by three vertices—boundary points—P_A_, P_B,_ and P_C_, which form an isosceles right-angled triangle (Figure 7a; some tolerances must be taken into account with respect to possible geometric deformations of a Data Matrix code). However, the initial position of the line segments P_A_-P_B_ and P_B_-P_C_ may not be optimal.

Minimizing the distance of the boundary line points to the candidate region points.

The optimal position of the boundary line P_A_-P_B_ can be defined as such, where the moment of inertia of the candidate region points is minimal, where line P_A_-P_B_ is an axis of rotation.

1. Start with an initial estimate of the boundary line P_A_-P_B_ and the five points wide region of interest along the P_A_-P_B_ boundary line (Figure 7b);
2. Calculate centroid *C* and gradient *k* of optimized boundary line *y* using raw and central statistical moments (when calculating the moments, take into account only the candidate region points which are in the region of interest):
  (4) $$C=\left(\frac{M_{10}}{M_{00}},\frac{M_{01}}{M_{00}}\right)=\left(C_{x},C_{y}\right),\;k=\frac{\mu_{02}-\mu_{20}+\sqrt{{\left(\mu_{20}-\mu_{02}\right)}^{2}+4\mu_{11}^{2}}\;}{2\mu_{11}},\;q=C_{y}-kC_{x},\;y=kx+q$$
3. Shift the end-points P_A_ and P_B_ of the boundary line using optimized line parameters *k* and *q*. If the orientation of the line *y* is vertical (|*k*| > 1) then use y-coordinates of the end-points P_A_ and P_B_ to update their x-coordinates (P_x_ = (P_y_ − *q*)/*k*), otherwise use x-coordinates to update their y-coordinates (P_y_ = P_x_ × *k* + *q*).Narrow the width of the region of interest by 1 point and repeat step 2;
4. Check if there is at least a 90% overlap of boundary line P_A_-P_B_ and the candidate region. If sufficient overlap is not found, the candidate region is rejected.

Apply the same procedure for the line segment P_B_-P_C_.

Processing continues in Section 2.3.

### 2.2. Adaptive Thresholding Methods (Method Group 2)

#### 2.2.1. Binarization Using Adaptive Thresholding

**Figure 8:**
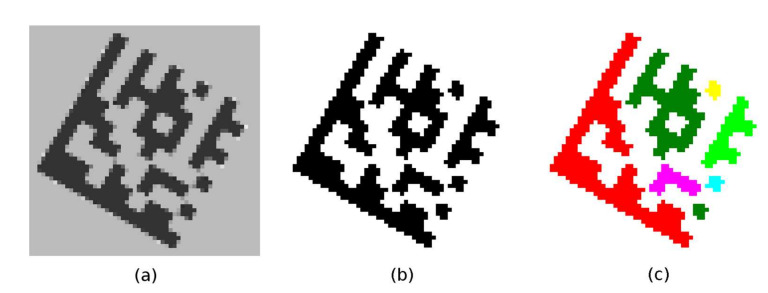
(**a**) grayscale image; (**b**) binarized image; (**c**) colored continuous regions.

Based on the assumption that the Data Matrix code is a darker object relative to its immediate surroundings, the adaptive thresholding technique is used. The expected result is that the points belonging to the dark modules become foreground points and the points belonging to the light modules become background points (Figure 8b). Adaptive thresholding techniques are able to distinguish dark and light points even when the image is unevenly illuminated. For each point in the image, an individual threshold *T*(*x*, *y*) is calculated, which is based on the intensity values of the points around it. In addition to the local mean *m*(*x*, *y*) (Equation (5)), local variance *s*^2^(*x*, *y*) (Equations (6) and (7)) is often used [10,11,12].

(5) $$T(x,y)=m(x,y)-delta\;m(x,y)=\frac{1}{w^{2}}\sum_{i=x-w/2}^{x+w/2}\sum_{j=y-w/2}^{y+w/2}I(i,j)$$

(6) $$T(x,y)=m(x,y)+ks(x,y)\;s^{2}(x,y)=\frac{1}{w^{2}}\sum_{i=x-w/2}^{x+w/2}\sum_{j=y-w/2}^{y+w/2}I{\left(i,j\right)}^{2}-m{\left(x,y\right)}^{2}$$

(7) $$T(x,y)=m(x,y)\left[1+k\left(\frac{s(x,y)}{128}-1\right)\right]$$

where *w* is a side of the local window with the center at (*x*, *y*) and *k* is constant. The size of the local window must correspond to the expected maximum module sizes (it should be at least five times greater; in our experiments, we worked with windows size 35 points).

Besides these well-known adaptive thresholding techniques, we also introduced our own (Equation (8)), which provided slightly better results:

(8) $$T(x,y)=m(x,y)-\frac{I(x,y)}{k_{1}}-\frac{s^{2}(x,y)}{k_{2}}$$

where *k*_1_ is a constant controlling penalization of light points, *k*_2_ is a constant controlling decreasing of the local threshold for points in which neighborhood intensity significantly varies (in our experiments *k*_1_ was set to 10 and *k*_2_ to 120).

#### 2.2.2. Connecting Foreground Points into Continuous Regions

The connected component labeling algorithm [9] is applied to the binary image obtained in the previous step. A region descriptor is maintained for each continuous region (BLOB). As the individual foreground points are added to the given continuous region, it is updated as follows:

- *Area* ← *Area* + 1;
- (*x*, *y*) coordinates of the added point are used to update 8 boundary points: *Top*-*Left*, *Top*-*Right*, *Right*-*Top*, *Right*-*Bottom*, *Bottom*-*Right*, *Bottom*-*Left*, *Left*-*Bottom*, *Left*-*Top.*

The result of the labeling algorithm is an image of the labels (each continuous region is assigned its number; Figure 8c) and at the same time each region is described by an area (number of foreground points) and a bounding octagon (defined by 8 boundary points).

(Note that this is the same procedure as in Section 2.1.2 with the difference that here the foreground points from the binary image are connected, while in Section 2.1.2 the edge points were connected).

#### 2.2.3. Evaluation of Finder Pattern Candidates

Region descriptors computed during the connected component labeling phase are used to filter out regions that cannot represent part of the Data Matrix code (including Finder Pattern). Considering that the Data Matrix code has a square shape, the area descriptors must meet the following conditions:

- Area of the region must be greater than 80 points (removes small areas, which cannot be Finder Pattern part of the Data Matrix code);
- Aspect Ratio (width to height ratio) of the region must be in the interval <0.5; 2>;
- Extent (ratio of the area of the region to the area of the outer bounding box) must be in the interval (0.1; 0.6).

**Figure 9:**
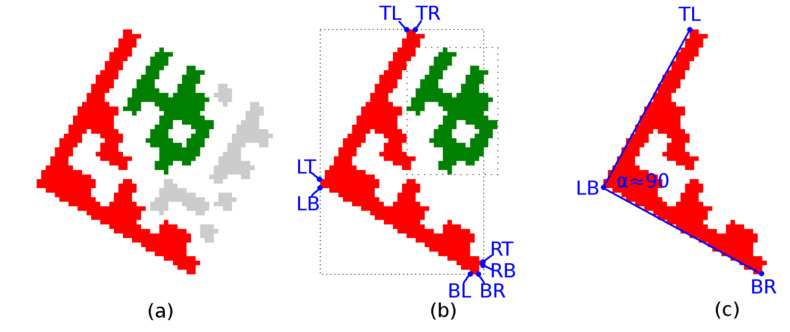
Data Matrix Finder Pattern candidates. (**a**) suppression of unfitting regions; (**b**) 8 boundary points of region; (**c**) 3 vertices of a right triangle.

These quick checks remove false-positive candidates (Figure 9a) but are not sufficient to identify true Data Matrix Finder Pattern candidates, so additional filtering is required.

Each candidate region is also described by 8 boundary points (Figure 9b) denoted as TL (Top-Left), TR (Top-Right), RT (Right-Top), RB (Right-Bottom), BR (Bottom-Right), BL (Bottom-Left), LB (Left-Bottom), LT (Left-Top). If the candidate region should be a Finder Pattern, then at least three points must form the vertices of an isosceles right-angled triangle (Figure 9c). The boundary points form two quadrilaterals, defined by four points:

- TL (Top-Left)–RT (Right-Top)–BR (Bottom-Right)–LB (Left-Bottom);
- TR (Top-Right)–RB (Right-Bottom)–BL (Bottom-Left)–LT (Left-Top).

**Figure 10:**
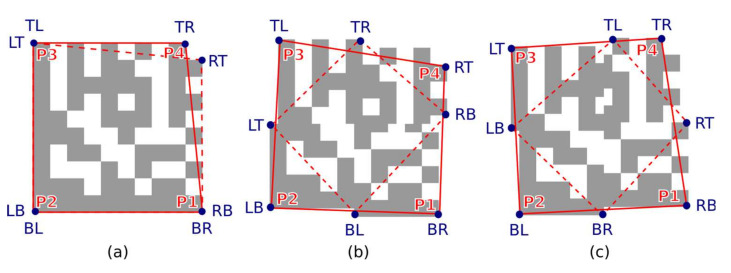
Outer bounding boxes of candidate regions. (**a**) unrotated code; (**b**) rotated clockwise; (**c**) rotated counterclockwise.

A quadrilateral with a bigger perimeter (the one that represents the outer boundary of the candidate region) is selected. As shown in Figure 10, the rotation of the Data Matrix code affects the selection of the outer quadrilateral P1-P2-P3-P4 (the solid red line indicates the outer quadrilateral, while the dashed line connects the boundary points that do not form the outer quadrilateral).

All four interior angles of the quadrilateral are evaluated to see whether their adjacent vertices can form the vertices of an isosceles right-angled triangle (Equation (9); the length of the two legs and the hypotenuse of the triangle is checked). Due to possible perspective distortion of the Data Matrix code, some tolerances must be considered:

(9) $$min(\left|P_{A},P_{B}\right|,\left|P_{B},P_{C}\right|)>28\;\left|\left|P_{A},P_{B}\right|-\left|P_{B},P_{C}\right|\right|<16\;\left|\left|P_{A},P_{C}\right|-\sqrt{{\left|P_{A},P_{B}\right|}^{2}+{\left|P_{B},P_{C}\right|}^{2}}\right|<\max(4,0.11\left|P_{A},P_{C}\right|)$$

where *P_A_*, *P_C_* are adjacent vertices of one of the interior angles *P_B_*.

If formula Equation (10) applies then the vertices are arranged counter-clockwise so that *P_A_* is top-left, P_B_ is bottom-left and P_C_ is the bottom-right vertex of the Finder Pattern [13]. In this case, the vertices *P_A_* and *P_B_* are swapped so that the vertices are arranged clockwise.

(10) $$\left(P_{A}.x-P_{B}.x\right)\left(P_{C}.y-P_{B}.y\right)-\left(P_{A}.y-P_{B}.y\right)\left(P_{C}.x-P_{B}.x\right)<0$$

(Note: Experiments have shown that if the 8 boundary points do not form a right-angled triangle, it is effective to shrink the candidate region by one point, update the boundary points and repeat the search again (small protrusions may appear on the edge of the Finder Pattern due to imperfect thresholding).

#### 2.2.4. Validating Finder Pattern Candidates and Aligning to Finder Pattern

The candidates for the Finder Pattern which have been identified on the basis of the relative position of the three vertices P_A_, P_B,_ and P_C_ must be verified and the position of the three vertices must be optimized to align to the Finder Pattern of the Data Matrix code in the image. Points P_A_, P_B_, P_C_ are the boundary points of the continuous region, but their position may not be optimal due to image noise, imperfect local thresholding, or defects in the Finder Pattern.

The following two alternative algorithms optimize the position of the boundary lines P_A_-P_B_ and P_B_-P_C_ so that they are aligned to the edge of the Finder Pattern.

##### Alternative 1: Approaching the Line Segment to the Region

The end-points of the line segment P_A_-P_B_ are alternately approached to the candidate region until at least a 90% overlap is achieved.

**Figure 11:**
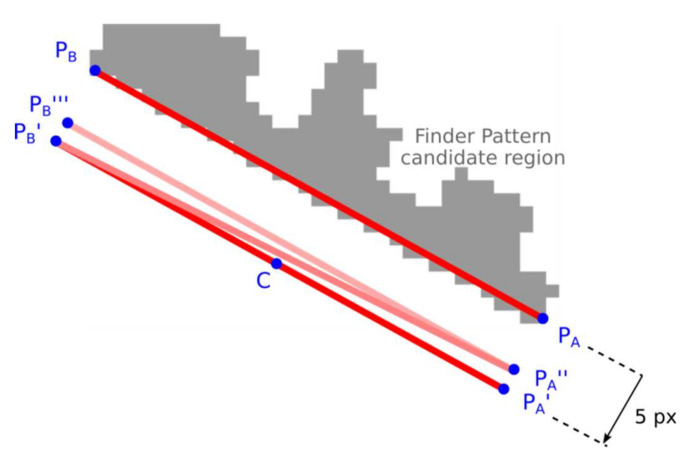
Approaching the line segment to the region.

1. Shift the initial estimate of the line segment P_A_-P_B_ by five points in the perpendicular direction away from the candidate region (Figure 11; P_A_’-P_B_’);
2. Calculate the overlap of line segments P_A_-C and C-P_B_ (C is the center of line segment P_A_-P_B_). Shift by one point, towards the candidate region, the end of the line segment that has less overlap. If there is no overlap, shift P_A_ and P_B_ alternately (zick-zack);
3. Stop approaching if 90% overlap is found. If no overlap is found after the specified number of iterations, the candidate region is rejected.

##### Alternative 2: Projections along the Line Segments

**Figure 12:**
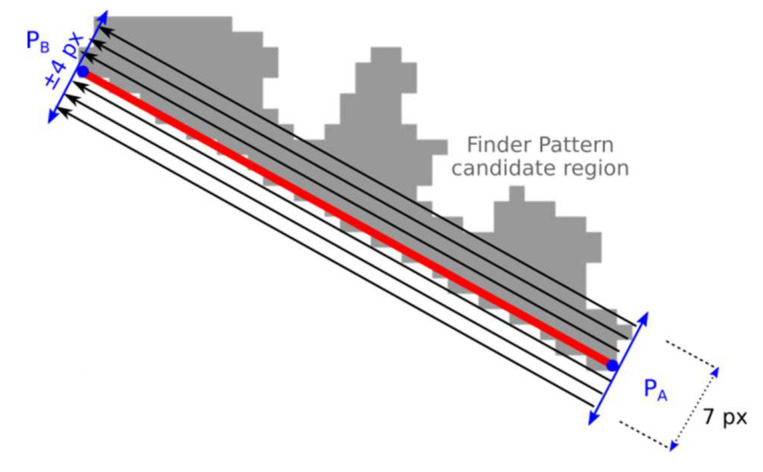
Projection along the line segment.

The optimal position of the end-points of the line segment P_A_-P_B_ (and similar line segment P_B_-P_C_) is such where the difference of adjacent projections is maximal—the edge is the strongest one (Figure 12).

1. Calculate projection *Proj* in the rectangular area along the line segment defined by end-points P_A_-P_B_. The width of the rectangular area is seven points and the length is |P_A_, P_B_|. For the purposes of calculating the projection (sum), the point that lies in the candidate region has a value of 1 otherwise 0. These values are summed and two adjacent values in projection *Proj* with the maximal difference are identified (*argmax*(*Proj*[*i* + 1] − *Proj*[*i* − 1]));
2. The points P_A_, P_B_ are independently shifted in both directions and step 1 is repeated for each shift. The position of the maximal difference is stored;
3. The optimal position of P_A_-P_B_ is such, where at least 80% of points of the line segment P_A_-P_B_ lie in the Finder Pattern candidate region, and at the same time, at least 40% of points are such that they lie in the candidate region while their adjacent points are outside the candidate region. If such a position is not found then the candidate region is rejected.

### 2.3. Identification of Perspective Distortion and Setting-Up Perspective Transformation

**Figure 13:**
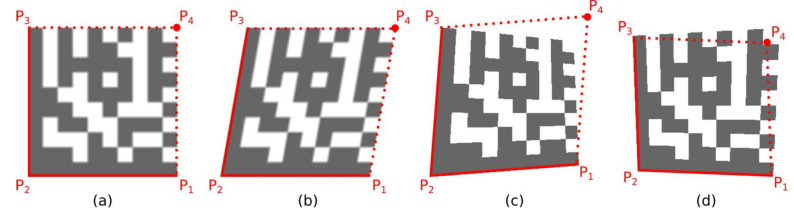
The initial estimate of the position of 4th point P_4_. (**a**) undistorted Data Matrix code; (**b**) skewed Data Matrix code; (**c**,**d**) perspective distorted Data Matrix codes.

The Finder Pattern candidate region is described by three vertices P_A_ (P_1_), P_B_ (P_2_), and P_C_ (P_3_). If perspective distorted Data Matrix codes are considered, the position of 4th point P_4_ must be determined. The initial estimate of the position of point P_4_ is obtained by extending to the parallelogram. As shown in Figure 13 this estimate must be corrected for perspective distorted Data Matrix codes.

#### 2.3.1. Evaluation Distance to Timing Pattern

**Figure 14:**
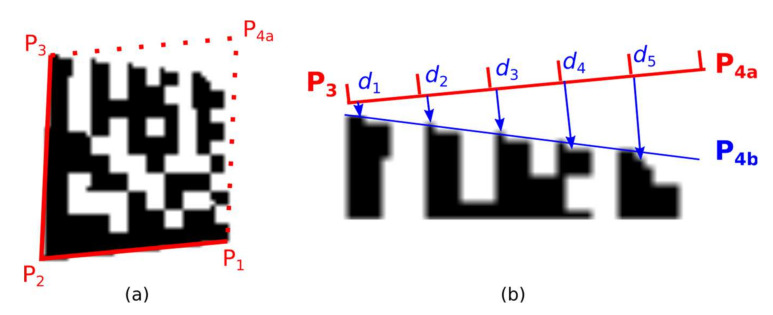
Perspective distorted Data Matrix code. (**a**) the initial estimate of the position of point P_4_; (**b**) distances from line segment P_3_-P_4a_ to Timing Pattern of Data Matrix code.

The first method of how to find the correct position of the fourth corner point P_4_ is based on moving the boundary lines P_3_–P_4_ and P_1_–P_4_ and evaluating the overlap of the boundary lines with a Data Matrix code candidate (Figure 14; Data Matrix code candidate area is defined by four points P_1_-P_2_-P_3_-P_4_).

The boundary line P_3_-P_4_ is shifted by two points away from the Data Matrix code candidate. The boundary line P_3_–P_4_ is divided into five slots. For each slot the minimal distance *d*_i_ to the nearest inner dark point of Data Matrix code candidate (Figure 14b) is computed and the number of dark points intersected by the boundary line.

- If *d*_5_ − *d*_1_ > 1 and *d*_i_ ≤ *d*_i+1_ then point P_4_ is shifted inward to the Data Matrix candidate area (a situation where P4 is located outside);
- If *d*_4_ − *d*_1_ < 0 and *d*_i_ ≥ *d*_i+1_ and there is at least one slot where there is 40% of intersected dark points, then P_4_ is shifted outward from the Data Matrix candidate area (a situation where P_4_ is located inside), and the procedure repeats.

#### 2.3.2. Setting-Up Perspective Transformation

**Figure 15:**
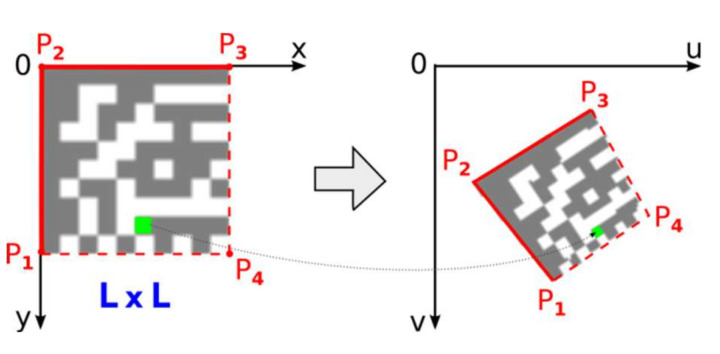
Perspective transformation.

Once the position of the 4th point, P_4_, is refined perspective transformation from the source square domain representing an ideal Data Matrix code to the quadrilateral destination representing a real Data Matrix code in the image can be set up (Figure 15).

Using the following equations [14]:

$$u=\frac{ax+by+c}{gx+hy+1},\;v=\frac{dx+ey+f}{gx+hy+1}$$

where coefficients can be computed from coordinates of four vertices P_1_(*u*_1_,*v*_1_), P_2_(*u*_2_, *v*_2_), P_3_(*u*_3_, *v*_3_), P_4_(*u*_4_, *v*_4_) as:

(11) $$a=\left(u_{3}-u_{2}\right)/L+gu_{3},\;b=\left(u_{1}-u_{2}\right)/L+hu_{1},\;c=u_{2}\;d=\left(v_{3}-v_{2}\right)/L+gv_{3},\;e=\left(v_{1}-v_{2}\right)/L+hv_{1},\;f=v_{2}\;g=\frac{\left|\begin{array}{cc} du_{3} & du_{2}\\ dv_{3} & dv_{2} \end{array}\right|}{\left|\begin{array}{cc} du_{1} & du_{2}\\ dv_{1} & dv_{2} \end{array}\right|},\;h=\frac{\left|\begin{array}{cc} du_{1} & du_{3}\\ dv_{1} & dv_{3} \end{array}\right|}{\left|\begin{array}{cc} du_{1} & du_{2}\\ dv_{1} & dv_{2} \end{array}\right|}\;du_{1}=(u_{3}-u_{2})L,\;du_{2}=(u_{1}-u_{4})L,\;du_{3}=u_{2}-u_{3}+u_{4}-u_{1}\;dv_{1}=(v_{3}-v_{2})L,\;dv_{2}=(v_{1}-v_{4})L,\;dv_{3}=v_{2}-v_{3}+v_{4}-v_{1}$$

### 2.4. Validating Data Matrix Code Area

The Data Matrix code area defined by four points P_1_-P_2_-P_3_-P_4_ is checked against the following criteria to further eliminate false candidate regions and thus reduce computational costs (at this point dimensions of a Data Matrix code (number of rows and columns) is not known so only relative metrics can be used). The Data Matrix code area is divided into four equally sized tiles, and for each tile, the following is checked:

- The density of black points inside the tile area is between 0.25 and 0.85 (laser engraved Data Matrix codes have a higher density of black points);
- The ratio of horizontal to vertical edge points inside the tile area is between 0.33 and 3.4.

In addition to the tiles, stricter criteria for the whole area are also checked:

- The density of black points inside the Data Matrix area is between 0.35 and 0.70;
- The ratio of horizontal to vertical edge points inside the Data Matrix area is between 0.75 and 1.25;
- The density of horizontal edge points is greater than five times the width and the density of vertical edge points is greater than five times the height.

A Data Matrix code creates an irregular chess-board-like structure where there is a relatively balanced number of black and white modules that form the edges at the same time.

### 2.5. Checking Timing Pattern of a Data Matrix Candidate

It is currently verified that the two sides of a Data Matrix candidate form an “L” shaped Finder Pattern. Subsequently, it must be verified that the two opposite sides form a Timing Pattern and the number of rows and columns must be determined. In the Timing Pattern, there are alternate dark and light modules.

#### 2.5.1. Checking Local Extremes along Expected Timing Patterns

**Figure 16:**
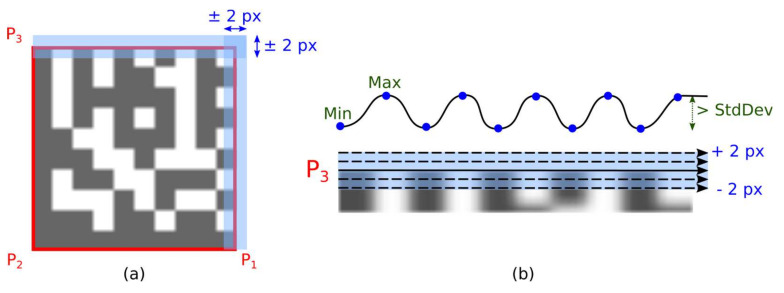
Local extremes in Timing Pattern areas. (**a**) Timing Pattern areas; (**b**) Local extremes.

The outer boundary of the Data Matrix candidate is defined by the four vertices P_1_, P_2_, P_3,_ and P_4_. However, the location of these corner points may not be completely accurate, so the Timing Pattern is examined in a wider area (blue rectangular areas along line segments P_3_-P_4_ and P_1_-P_4_ in Figure 16a).

Timing Pattern areas (vertical and horizontal) are scanned line by line. For each line scan are computed:

- number of transitions between dark and light modules (as a threshold value is used average gray-scale intensity in the Data Matrix candidate region);
- number of local extremes (blue dots in Figure 16b), that differs more than one standard deviation in gray-scale intensity in the Data Matrix candidate region);
- the sum of absolute values of gradients (changes in gray-scale intensities between two adjacent pixels);

If the number of local minima is the same as the number of local maxima then the number of local extremes determines the dimension (number of rows or columns) of the examined Data Matrix code. Otherwise, the number of transitions between dark and light modules is used to determine the dimensions of the Data Matrix code.

The determined dimension must be greater than 10 and must be even. If only square Data Matrix codes are considered, then the number of rows must be equal to the number of columns.

The optimal position of the boundary line is that where the sum of the gradients is maximum.

#### 2.5.2. Checking Horizontal and Vertical Edge Projections

**Figure 17:**
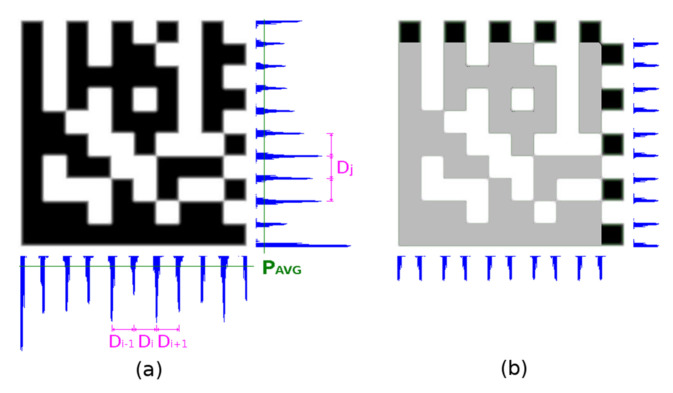
Horizontal and vertical edge projections of the Data Matrix code. (**a**) projections of the whole area; (**b**) projections of the Timing Pattern.

It sometimes happens that the Timing Pattern on any side of the Data Matrix code is damaged and the prior step gives a different result for the number of rows and columns. In this case, edge projections are used as a fallback method. Firstly, the edge projections of the whole area of the Data Matrix code candidate are evaluated (Figure 17a), secondly edge projections of the surrounding area of the Timing Pattern are evaluated (Figure 17b).

A histogram *H* of the distances between local maxima P_LMAX_ in edge projections is constructed. The distance with the highest occurrence *D*_M_ (mode value) is taken as the integer estimate of the module width. The real module width *MW* is calculated as the weighted average of D_M_ and two adjacent distances (Equation (12)):

(12) $$MW=\frac{\left(D_{M}-1)H[D_{M}-1]+D_{M}H[D_{M}]+(D_{M}+1)H[D_{M}+1\right]}{H[D_{M}-1]H[D_{M}]H[D_{M}+1]}$$

Local maxima P_LMAX_ in edge projections must meet one of the following criteria (P_AVG_ is average and P_STDDEV_ is a standard deviation of projection):

- It is local maxima on <−1, +1> and its edge projection value P_LMAX_ > P_AVG_ + ¼ P_STDDEV;_
- It is local maxima on <−2, +2> and its edge projection value P_LMAX_ > P_AVG_ − ½ P_STDDEV_ (if modules are blurred/eroded there can be small local maxima under average).

The averages, standard deviations, local maxima, and modulus sizes are calculated independently for horizontal and vertical projection.

The number of modules is then determined from the width of the Data Matrix code and the module width *MW* is rounded to an even number.

(Note: By maximizing the standard deviation in the edge projections, it is also possible to iteratively determine the position of P_4_ in case the Data Matrix code is perspective distorted. We introduced this approach for QR Codes in [15]).

### 2.6. Decoding the Data Matrix Code

**Figure 18:**
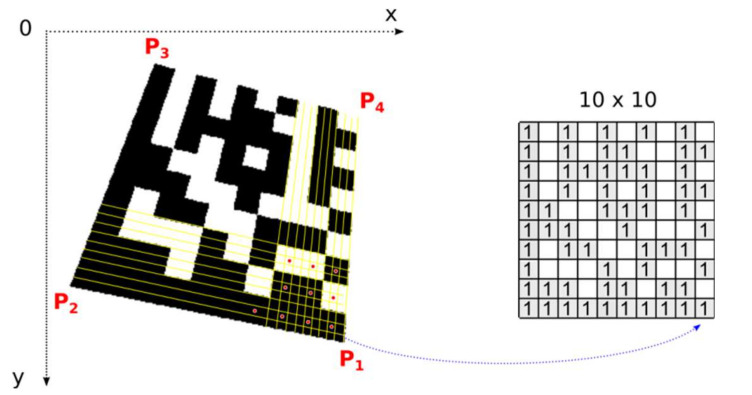
Transformation of the Data Matrix code from the image domain into the binary matrix.

Once the precise location of all four corner points, which form the bounding quadrilateral, is established, perspective transformation is set up and the number of rows and columns is determined, we can map image points from the Data Matrix code in the image to the square binary matrix (Figure 18). Dark modules must be mapped to binary 1 and light modules to binary 0. This binary matrix is the input of a deterministic process that decodes the data stored in the Data Matrix Code.

The open-source libdmtx library [16] is used to decode the binary matrix, which restores the original text encoded in the Data Matrix code.

One module in a real image is usually formed by a group of several points. One central point of the module decides whether the module is classified as dark or light (red points in Figure 18 represent the central points of the modules). Points with an intensity above the threshold are considered light, the others dark. The threshold is set as the average gray intensity in the Data Matrix code region. If the modules are made up of more than 5 × 5 points, we can include them in the decision-making, in addition to the central point, also points in its immediate surrounding (e.g., points forming a cross “+”).

## 3. Results

**Figure 19:**
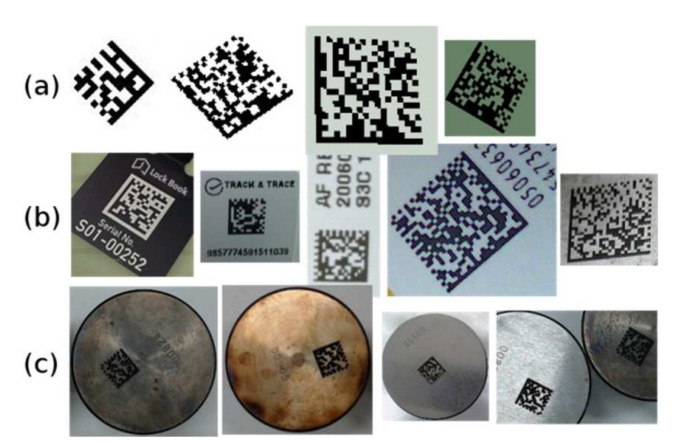
Testing samples: (**a**) synthetic; (**b**) Internet; (**c**) industrial.

The methods described above were compared on a test set consisting of 111 samples of Data Matrix codes. The testing dataset contained 21 synthetic Data Matrix codes of various sizes and rotations, 55 Data Matrix codes from Internet images, and 35 Data Matrix codes from a specific manufacturing process (marked by laser onto a metal surface). The samples of the test codes for these three groups are shown in Figure 19.

In Table 2 there are the compared results of the Data Matrix localization methods described in Section 2 (and also one another open-source solution is included). In the table, there are the numbers of successfully recognized/decoded Data Matrix codes (for individual groups of samples as well as for the whole test set consisting of a total of 111 codes).

**Figure 20:**
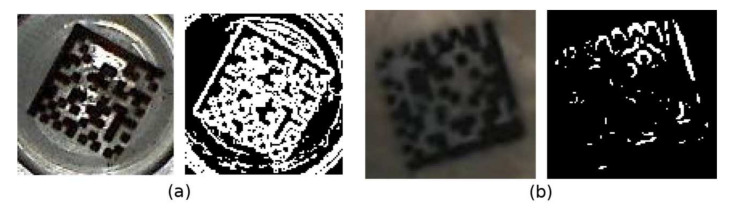
Edge detection problems: (**a**) Merged edges; (**b**) Missing edges.

As shown, methods based on adaptive thresholding (M3, M4) achieve better results than methods based on edge detection (M1, M2). This group of methods was also able to segment Data Matrix codes in low contrast images. (Note that, unlike other articles, we consider a Data Matrix code to be successfully recognized only if it is also decoded, not just localized). Edge detection methods that use a fixed threshold can determine a significant edge fail if there are reflections (false edges) near the Finder Pattern (Figure 20a) or there is low contrast between the Finder Pattern and the surroundings (Figure 20b).

In Table 3, the relative time consumptions of proposed methods are shown. Because the images in the test dataset have different resolutions and different numbers of Data Matrix codes in one image, we used the total test time for all the images in the test dataset to determine the overall time consumption (tested methods were implemented in Free Pascal without using any optimized computer vision libraries). Note that, all the presented methods differ in the initial steps—Finder Patter localization phase, and then share the next recognition steps.

Our testing dataset is published online together with this article under “Supplementary Material”.

As can be seen from the experimental results, the M3 method achieved the best recognition rate. Methods based on edge detection (M1, M2) had lower computational complexity, but at the cost of a lower recognition rate. Experiments have also shown that recognition rate can be further increased by:

- Appropriate pre-processing: by applying morphological conditional dilation with structuring element 3 × 3 to the binary image (replaces in the binary image non-object points that become object points after morphological closing), the nearby broken parts of the interrupted Finder Pattern are joined (small white areas are removed);
- Recognition in altered scale space: repeat recognition for the original image resized in scales 4:3 and 2:1 (Table 4; the side effect of scaling is smoothing the image).

## 4. Conclusions

We have designed and compared several methods for the localization of the 2D Data Matrix codes in arbitrary images under various lighting conditions. These methods are suitable for the localization of single or multiple Data Matrix codes in low-resolution images and are computationally undemanding. All the proposed methods use a typical “L” shaped Finder Pattern to identify the position of the Data Matrix codes in an image. Prerequisites of our methods are the existence of a relatively intact Finder Pattern and a quiet zone around a Data Matrix code.

This comparative study summarizes various approaches usable in recognizing Data Matrix codes, which provides the reader with a comprehensive review of the topic.

The proposed methods were validated on the testing set consisting of synthetic and also real-world samples. The experimental results show that our methods have a great detection rate.

Data Matrix codes as automatic identification and data capture (AIDC) technology can be used to mark transport units in logistics, parts in production, warehouse positions, sold goods and can also be used in automated robot navigation in production engineering [17,18,19,20,21].

## Figures and Tables

**Table 1:** Comparison of Data Matrix codes and QR codes.

Feature	Data Matrix Codes	QR Codes
Size	from 10 × 10 to 144 × 144 modules (increment by +2)	from 21 × 21 to 177 × 177 modules (incremented by +4)
Capacity	3116 numeric or 2335 alphanumeric or 1556 bytes	7089 numeric or 4296 alphanumeric or 2953 bytes (at lowest error correction level)
Error Correction Level	one level up to 30% damage	four configurable levels: L (Low): 7%, M (Medium): 15%, Q (Quartile): 25%, H (High): 30%
Finder Pattern	“L” shaped on the edge	at three corners of a QR Code. Each Finder Pattern is formed by an inner dark square surrounded by a dark frame
Timing Pattern	alternating dark and light modules at the edge	alternating dark and light modules placed inside a QR Code and interconnecting Finder Patterns
License	public domain	public domain

**Table 2:** Recognition results for different localization methods.

Method Description	Synthetic Samples (a)	Internet Samples (b)	Industrial Samples (c)	All Samples (a) + (b) + (c)
libdmtx (open-source) [16]	15	53	28	96
**(M1)** Method described in Section 2.1.1 (edge detection, perpendicular linear regions)	21	48	19	88
**(M2)** Method described in Section 2.1.2, Section 2.1.3 and Section 2.1.4 (edge detection, right-angled triangle from region boundary points)	21	43	18	82
**(M3)** Method described in Section 2.2 + Alternative 1 (adaptive thresholding, right-angled triangle from region boundary points + aligning to the Finder Pattern by approaching line segment to region)	21	55	34	**110**
**(M4)** Method described in Section 2.2 + Alternative 2 (adaptive thresholding, right-angled triangle from region boundary points + aligning to the Finder Pattern using projections along Finder Pattern)	21	55	33	**109**

**Table 3:** Approximate relative time consumption of tested methods.

Method	Relative Time Consumption	Average Time Per Image
(M1)	88%	6.1 ms
(M2)	58%	4.0 ms
(M3)	100%	6.8 ms
(M4)	119%	8.1 ms

**Table 4:** Recognition results for repeated runs in multiple scales of images.

Method	Original Images in Scale 1:1	Rescaled Images in ‘’Scales 1:1, 1:0.75, 1:0.5
(M1)	88	100
(M2)	82	91
(M3)	110	111
(M4)	109	111

## Supplementary Materials

The following are available online at https://www.mdpi.com/article/10.3390/jimaging7090163/s1; one ZIP file contains, one ZIP file contains images of the testing dataset of the Data Matrix codes used to test our proposed methods.
