# Supplementary figures and images for: Comparative Study of Data Matrix Codes Localization and Recognition Methods
Source: J Imaging. 2021 Aug 27;7(9):163. doi: 10.3390/jimaging7090163 (PMC8471265; doi:10.3390/jimaging7090163)

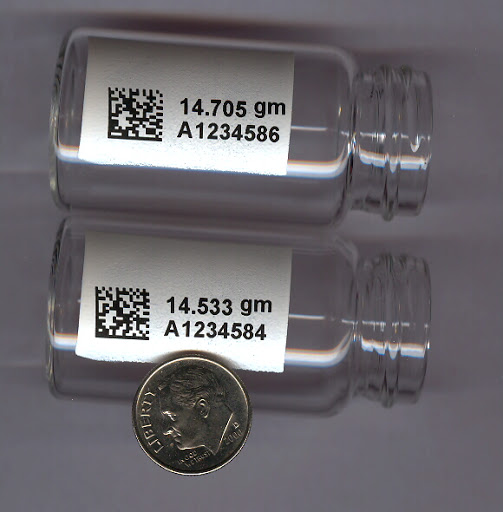

Supplement: Supplementary file 1 [file jimaging-07-00163-s001.zip › DMX6h.jpg]

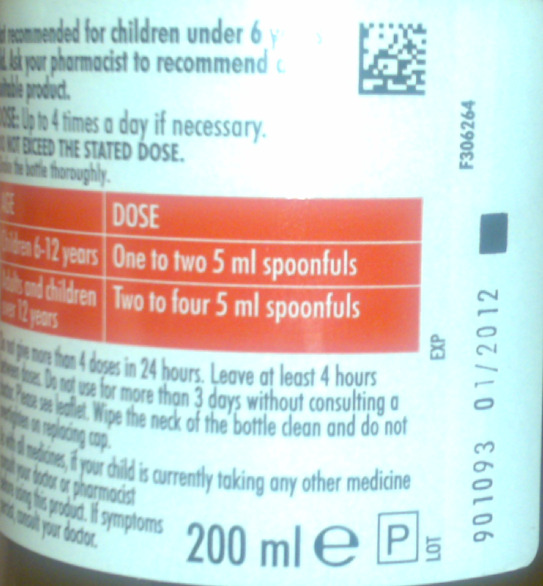

Supplement: Supplementary file 1 [file jimaging-07-00163-s001.zip › DMX5ae.jpg]

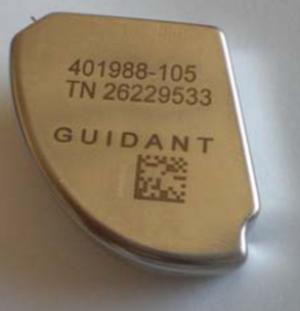

Supplement: Supplementary file 1 [file jimaging-07-00163-s001.zip › DMX5ad.jpg]

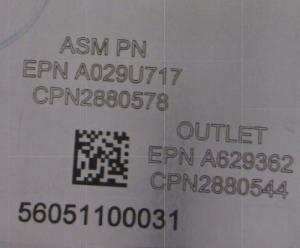

Supplement: Supplementary file 1 [file jimaging-07-00163-s001.zip › DMX5ac.jpg]

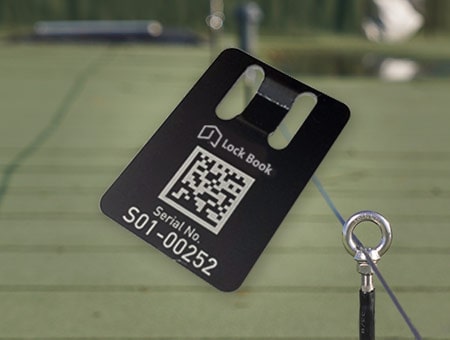

Supplement: Supplementary file 1 [file jimaging-07-00163-s001.zip › DMX5ab.jpg]

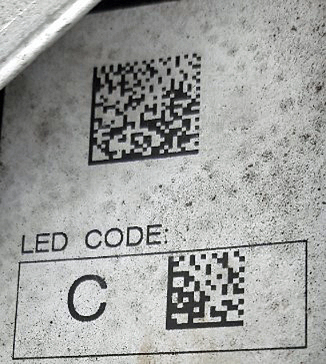

Supplement: Supplementary file 1 [file jimaging-07-00163-s001.zip › DMX6g.jpg]

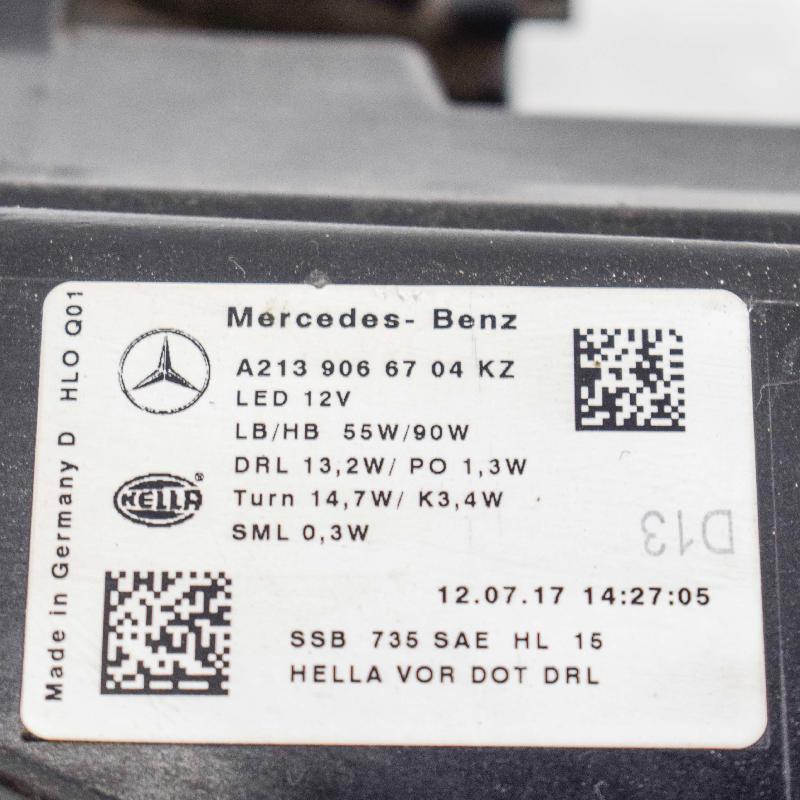

Supplement: Supplementary file 1 [file jimaging-07-00163-s001.zip › DMX5aa.jpg]

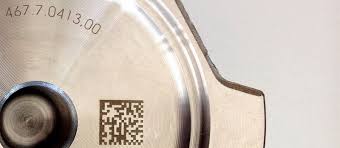

Supplement: Supplementary file 1 [file jimaging-07-00163-s001.zip › DMX5z.jpg]

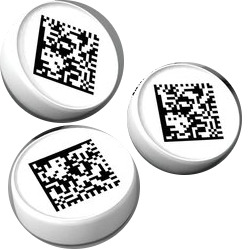

Supplement: Supplementary file 1 [file jimaging-07-00163-s001.zip › DMX6f.jpg]

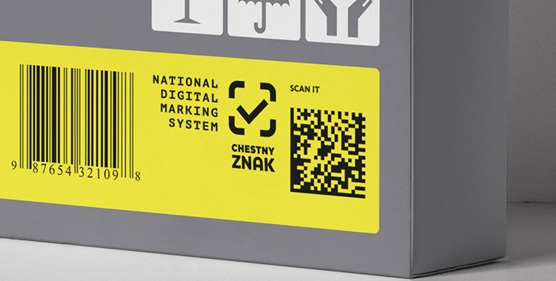

Supplement: Supplementary file 1 [file jimaging-07-00163-s001.zip › DMX6e.jpg]

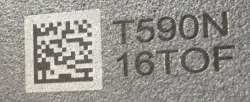

Supplement: Supplementary file 1 [file jimaging-07-00163-s001.zip › DMX5y.jpg]

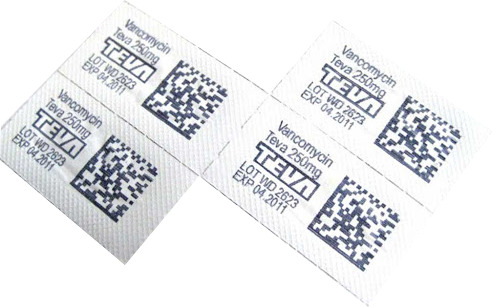

Supplement: Supplementary file 1 [file jimaging-07-00163-s001.zip › DMX5x.jpg]

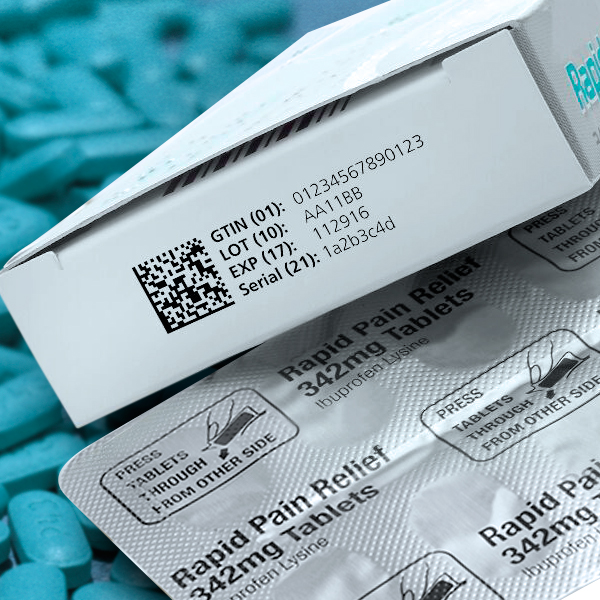

Supplement: Supplementary file 1 [file jimaging-07-00163-s001.zip › DMX6d.jpg]

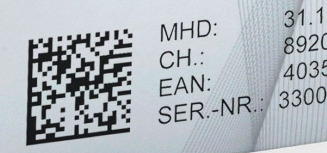

Supplement: Supplementary file 1 [file jimaging-07-00163-s001.zip › DMX6c.jpg]

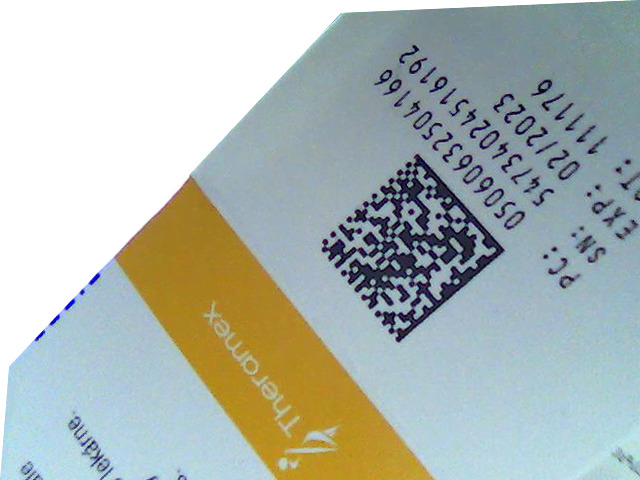

Supplement: Supplementary file 1 [file jimaging-07-00163-s001.zip › DMX6b.jpg]

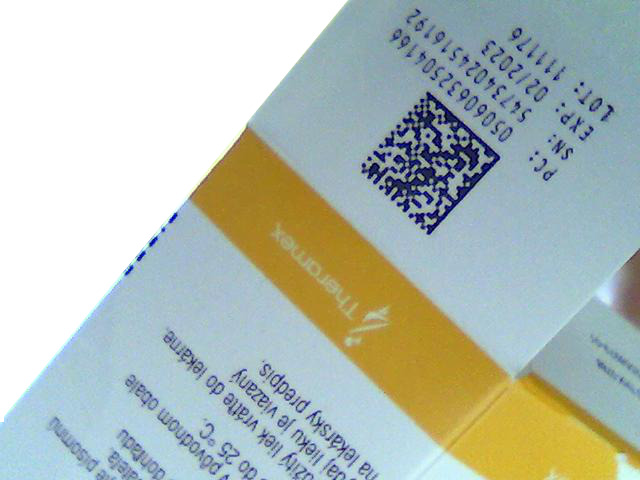

Supplement: Supplementary file 1 [file jimaging-07-00163-s001.zip › DMX5w.jpg]

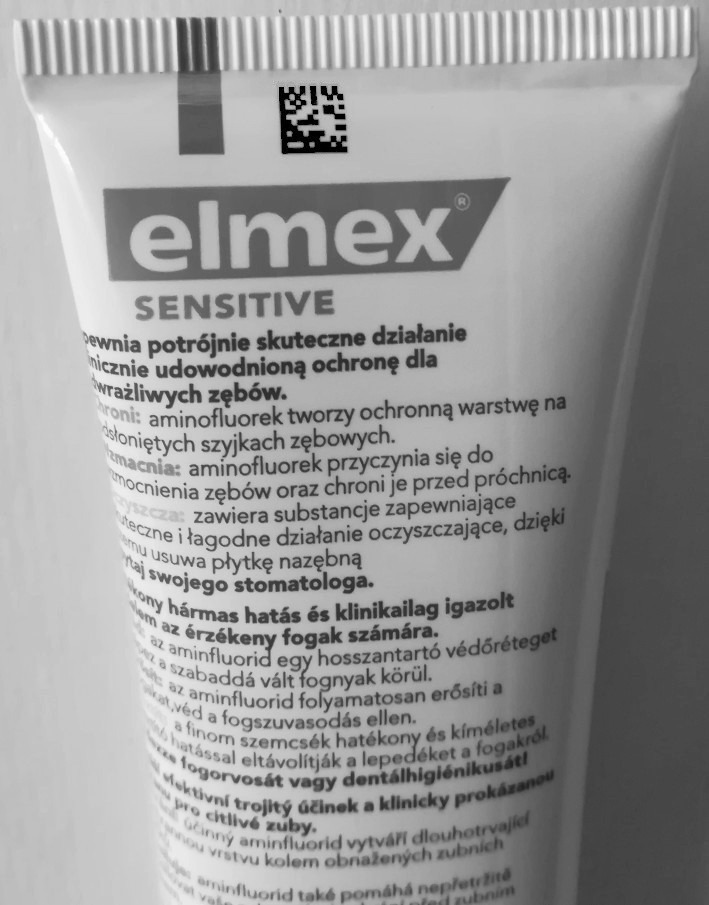

Supplement: Supplementary file 1 [file jimaging-07-00163-s001.zip › DMX5e.jpg]

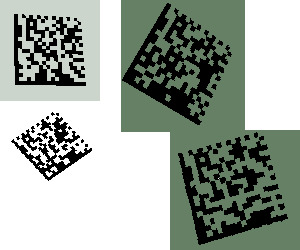

Supplement: Supplementary file 1 [file jimaging-07-00163-s001.zip › DMX6a.jpg]

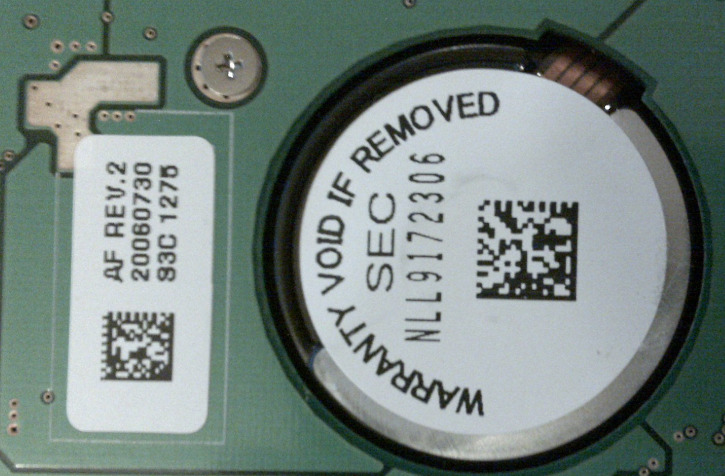

Supplement: Supplementary file 1 [file jimaging-07-00163-s001.zip › DMX5v.jpg]

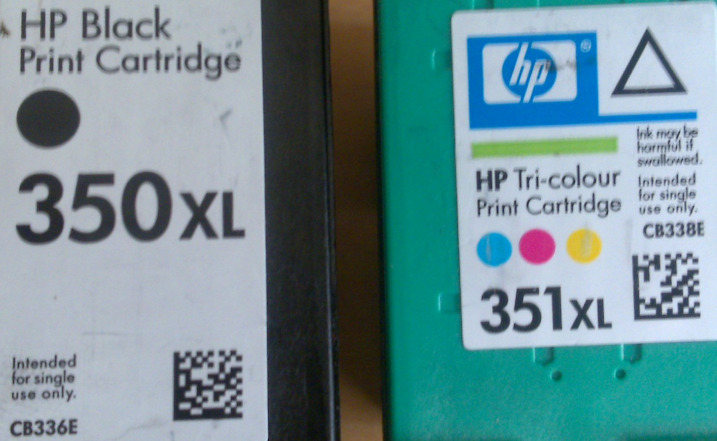

Supplement: Supplementary file 1 [file jimaging-07-00163-s001.zip › DMX5u.jpg]

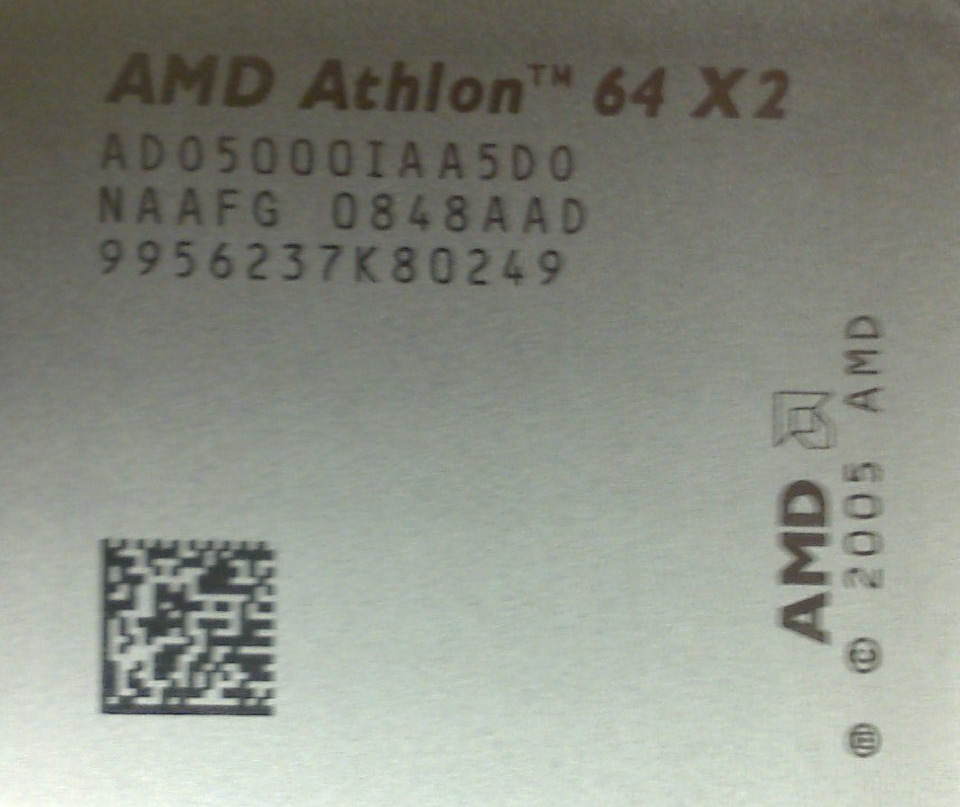

Supplement: Supplementary file 1 [file jimaging-07-00163-s001.zip › DMX5t.jpg]

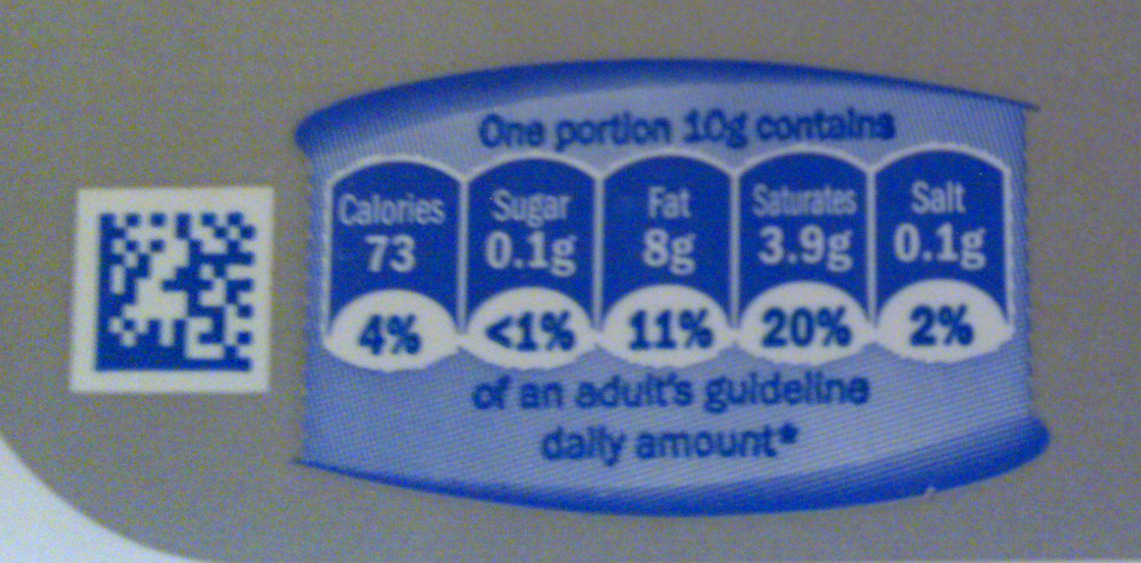

Supplement: Supplementary file 1 [file jimaging-07-00163-s001.zip › DMX5s.jpg]

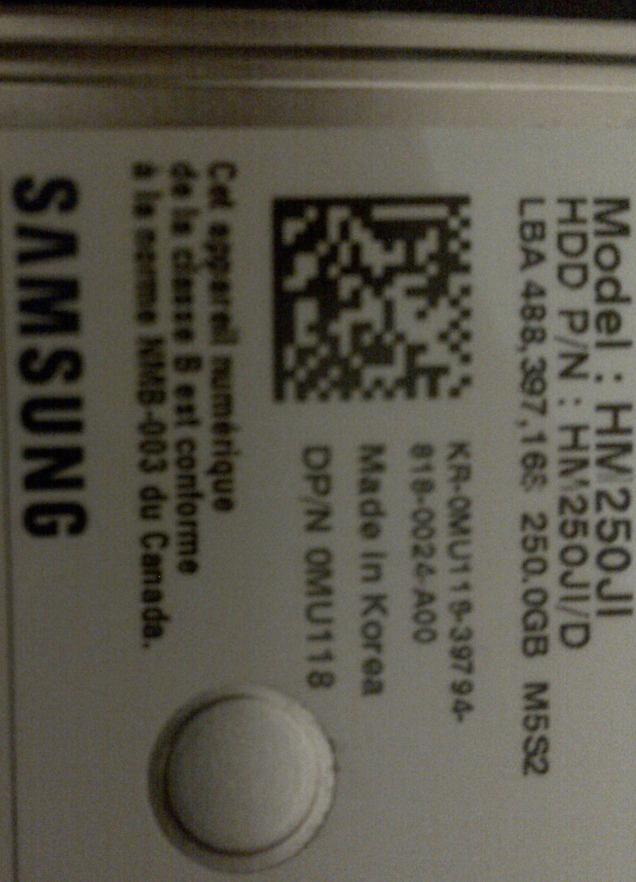

Supplement: Supplementary file 1 [file jimaging-07-00163-s001.zip › DMX5r.jpg]

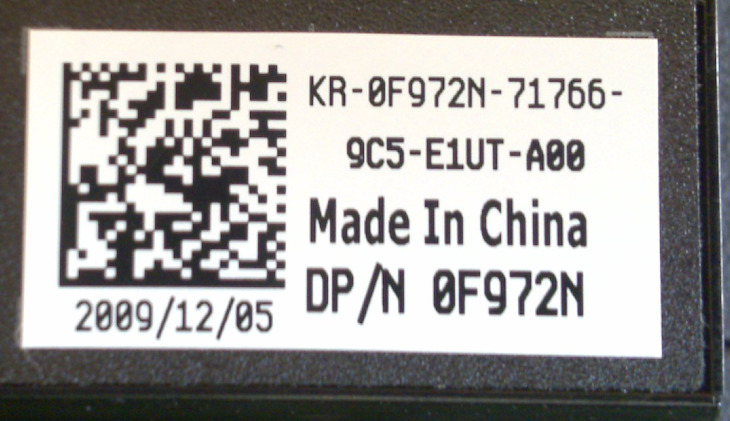

Supplement: Supplementary file 1 [file jimaging-07-00163-s001.zip › DMX5q.jpg]

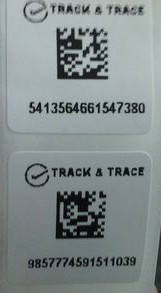

Supplement: Supplementary file 1 [file jimaging-07-00163-s001.zip › DMX5p.jpg]

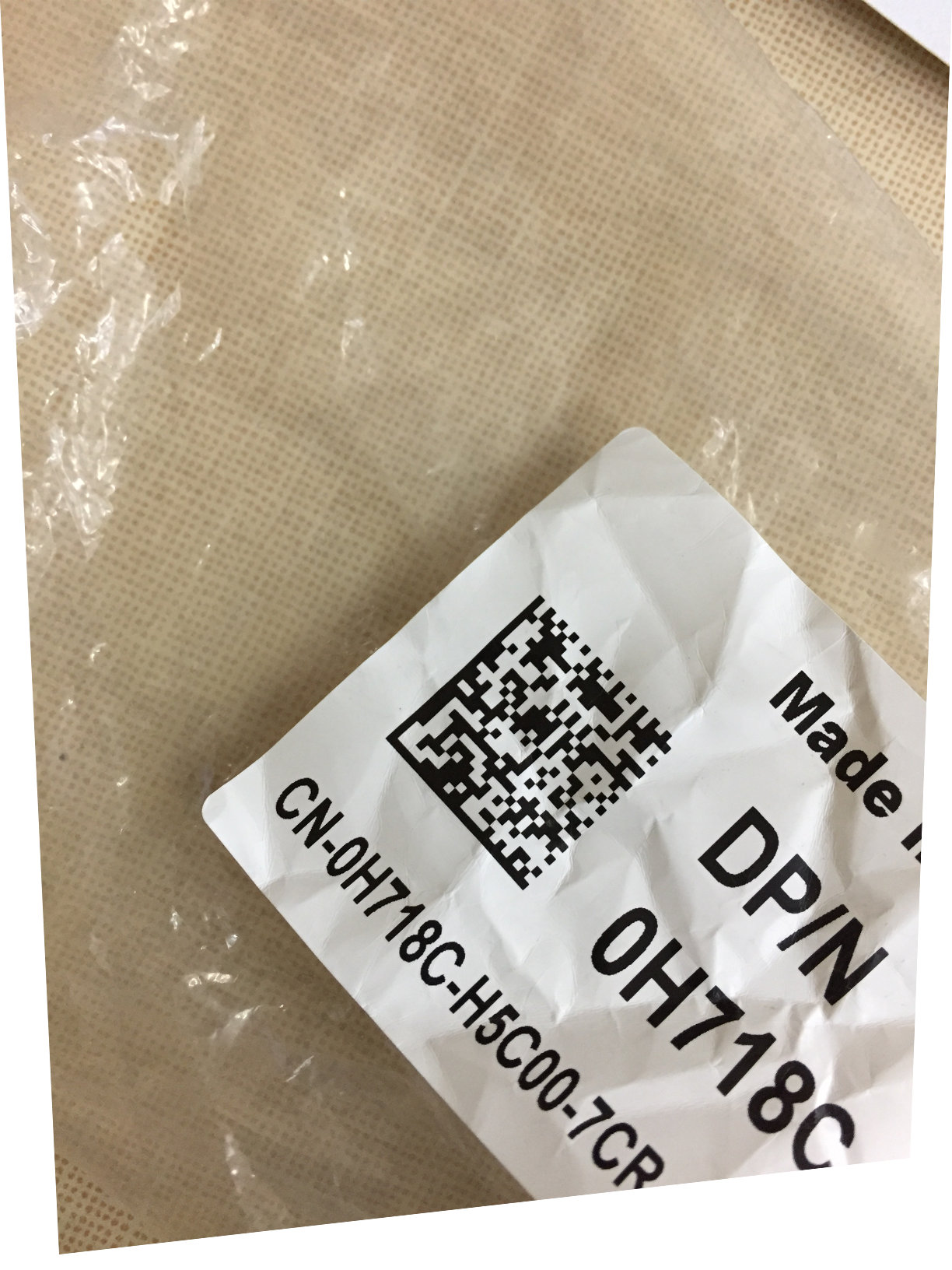

Supplement: Supplementary file 1 [file jimaging-07-00163-s001.zip › DMX5n.jpg]

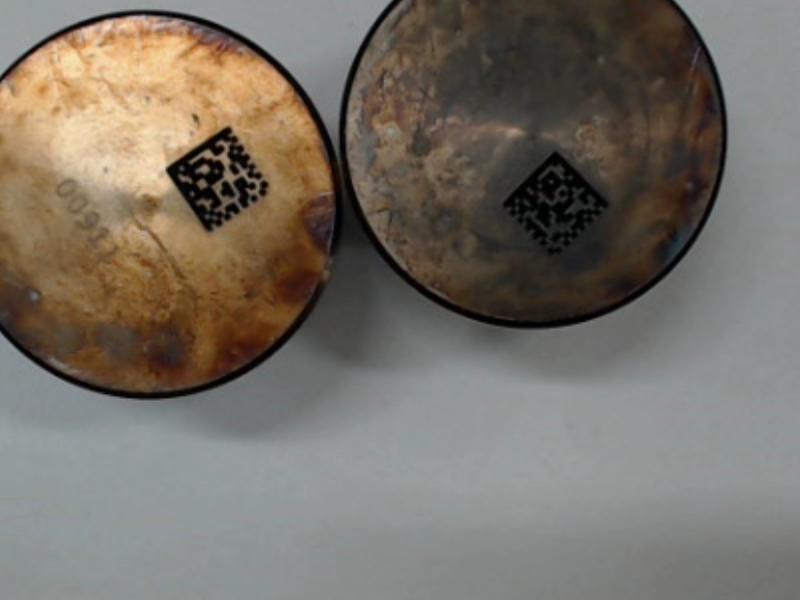

Supplement: Supplementary file 1 [file jimaging-07-00163-s001.zip › DMX2m.jpg]

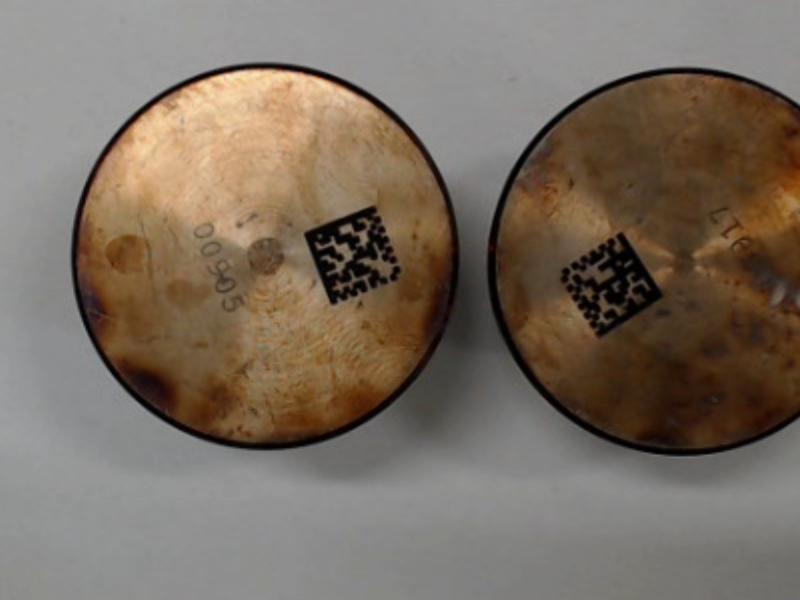

Supplement: Supplementary file 1 [file jimaging-07-00163-s001.zip › DMX2l.jpg]

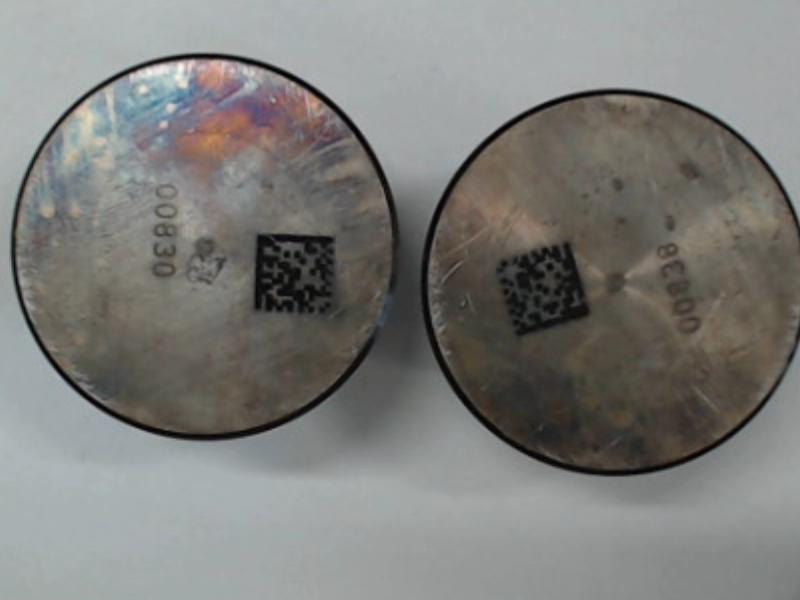

Supplement: Supplementary file 1 [file jimaging-07-00163-s001.zip › DMX2k.jpg]

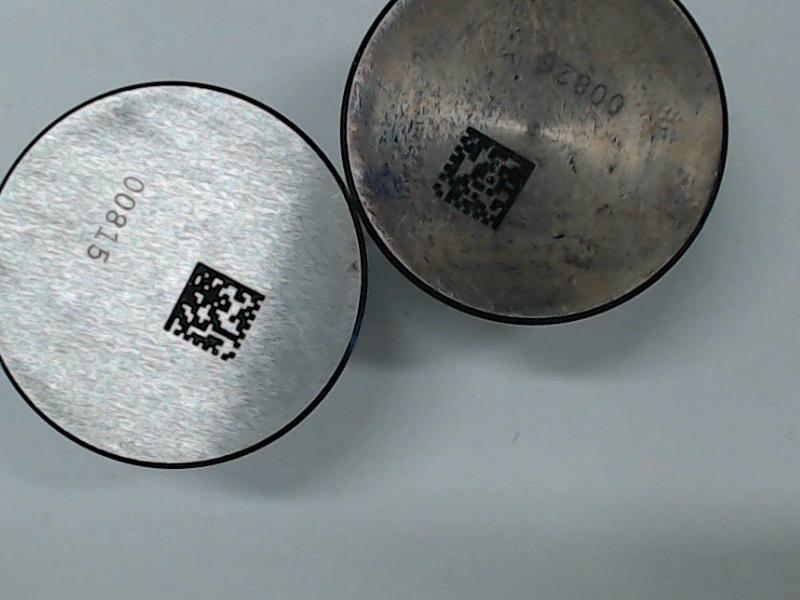

Supplement: Supplementary file 1 [file jimaging-07-00163-s001.zip › DMX2j.jpg]

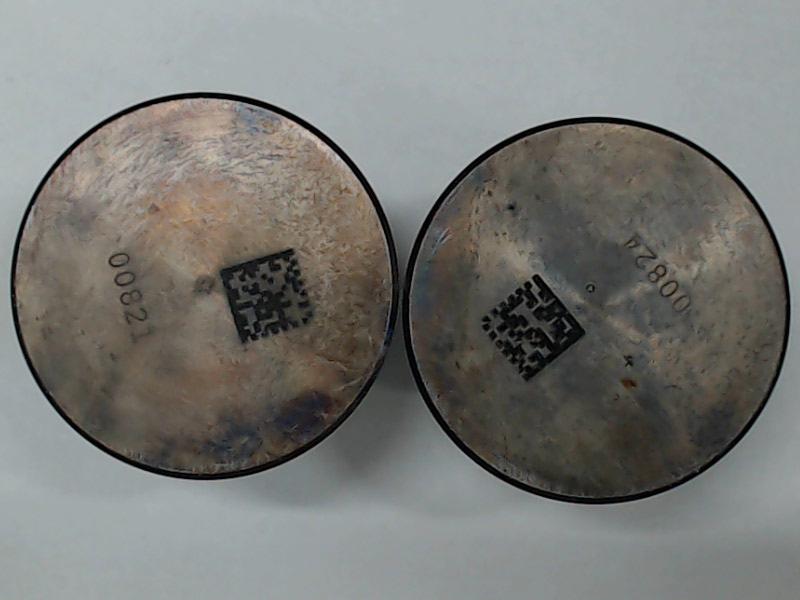

Supplement: Supplementary file 1 [file jimaging-07-00163-s001.zip › DMX2i.jpg]

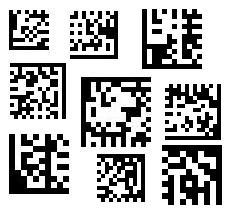

Supplement: Supplementary file 1 [file jimaging-07-00163-s001.zip › DMX1a.jpg]

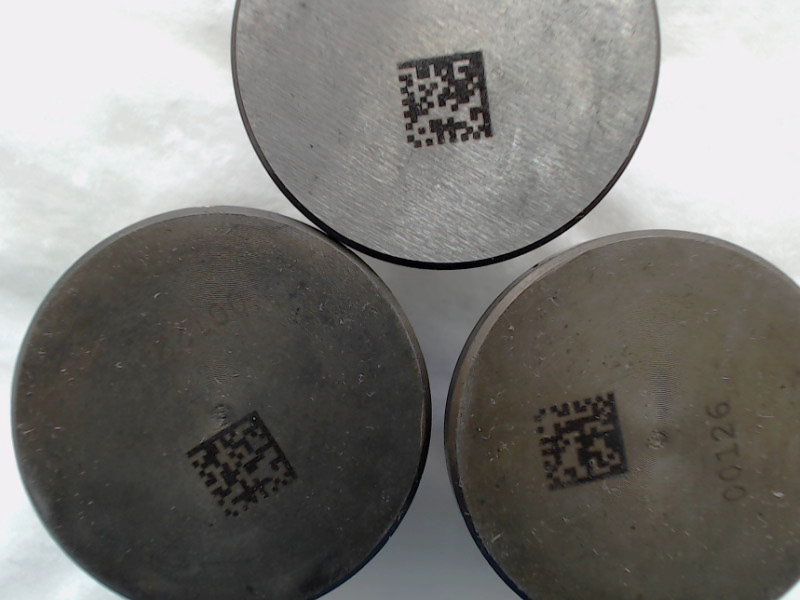

Supplement: Supplementary file 1 [file jimaging-07-00163-s001.zip › DMX2h.jpg]

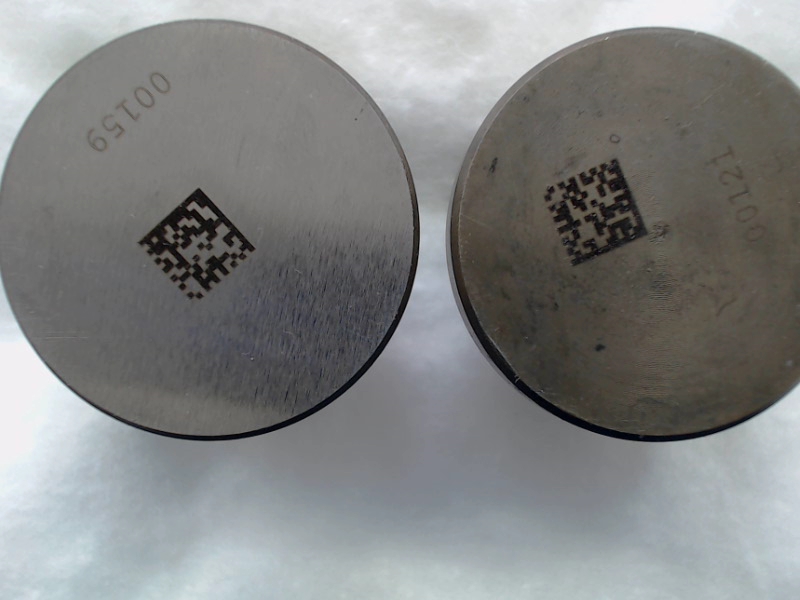

Supplement: Supplementary file 1 [file jimaging-07-00163-s001.zip › DMX2g.jpg]

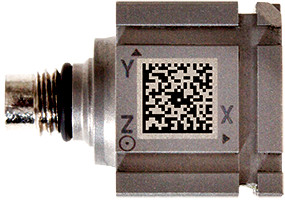

Supplement: Supplementary file 1 [file jimaging-07-00163-s001.zip › DMX5m.jpg]

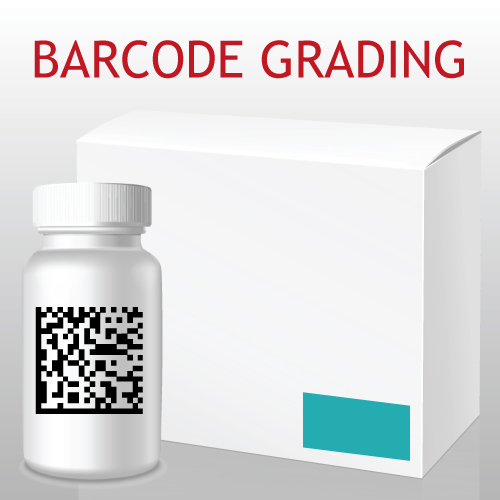

Supplement: Supplementary file 1 [file jimaging-07-00163-s001.zip › DMX5l.jpg]

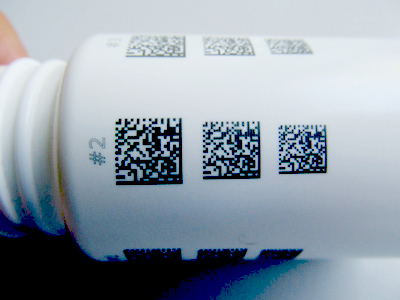

Supplement: Supplementary file 1 [file jimaging-07-00163-s001.zip › DMX5k.jpg]

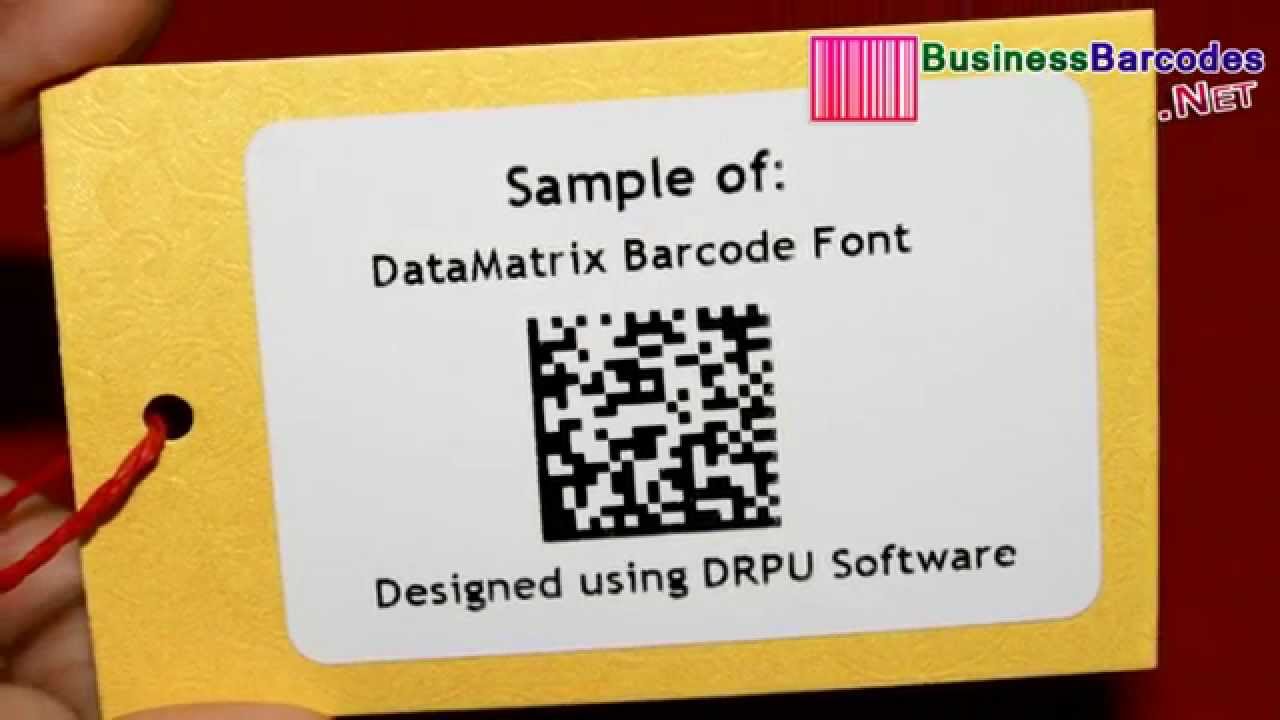

Supplement: Supplementary file 1 [file jimaging-07-00163-s001.zip › DMX5j.jpg]

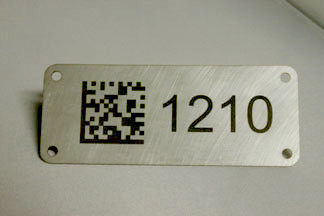

Supplement: Supplementary file 1 [file jimaging-07-00163-s001.zip › DMX5i.jpg]

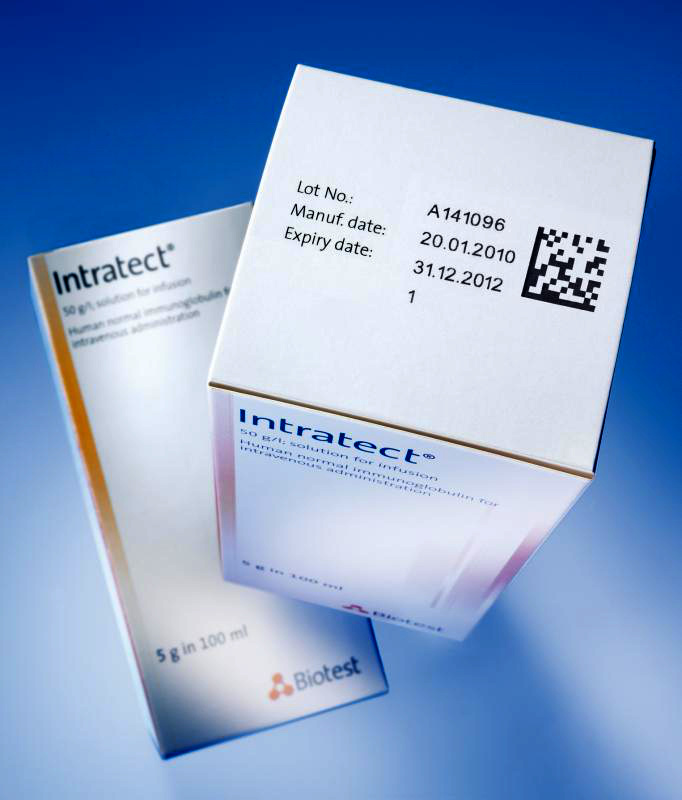

Supplement: Supplementary file 1 [file jimaging-07-00163-s001.zip › DMX5o.jpg]

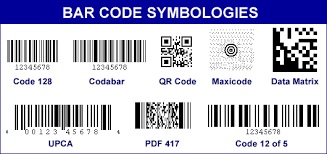

Supplement: Supplementary file 1 [file jimaging-07-00163-s001.zip › DMX5h.jpg]

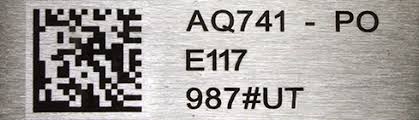

Supplement: Supplementary file 1 [file jimaging-07-00163-s001.zip › DMX5g.jpg]

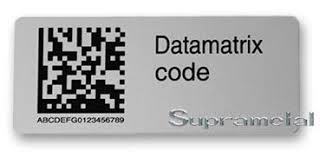

Supplement: Supplementary file 1 [file jimaging-07-00163-s001.zip › DMX5f.jpg]

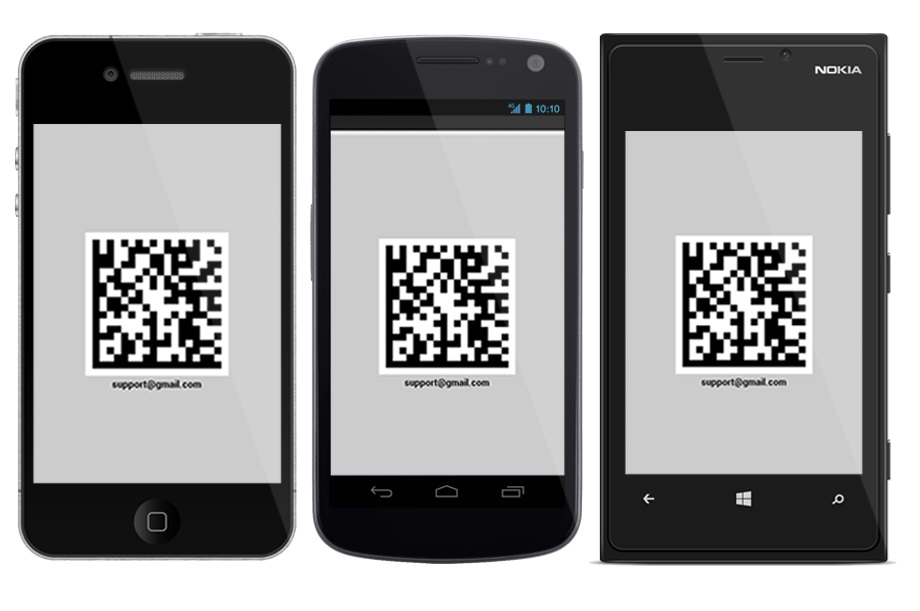

Supplement: Supplementary file 1 [file jimaging-07-00163-s001.zip › DMX5d.jpg]

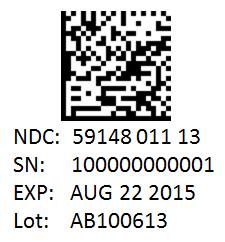

Supplement: Supplementary file 1 [file jimaging-07-00163-s001.zip › DMX5c.jpg]

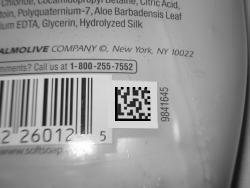

Supplement: Supplementary file 1 [file jimaging-07-00163-s001.zip › DMX5b.jpg]

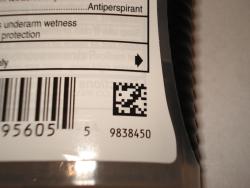

Supplement: Supplementary file 1 [file jimaging-07-00163-s001.zip › DMX5a.jpg]

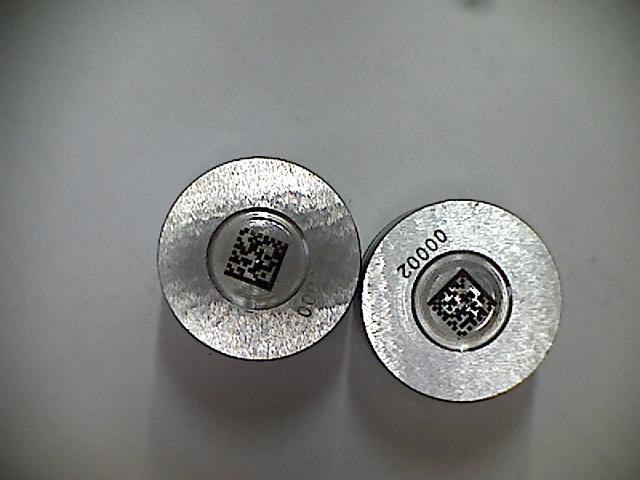

Supplement: Supplementary file 1 [file jimaging-07-00163-s001.zip › DMX3c_small.jpg]

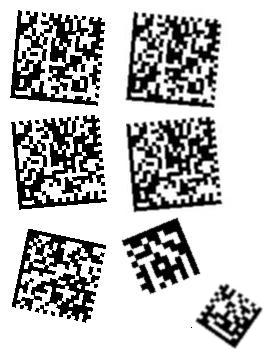

Supplement: Supplementary file 1 [file jimaging-07-00163-s001.zip › DMX1c.jpg]

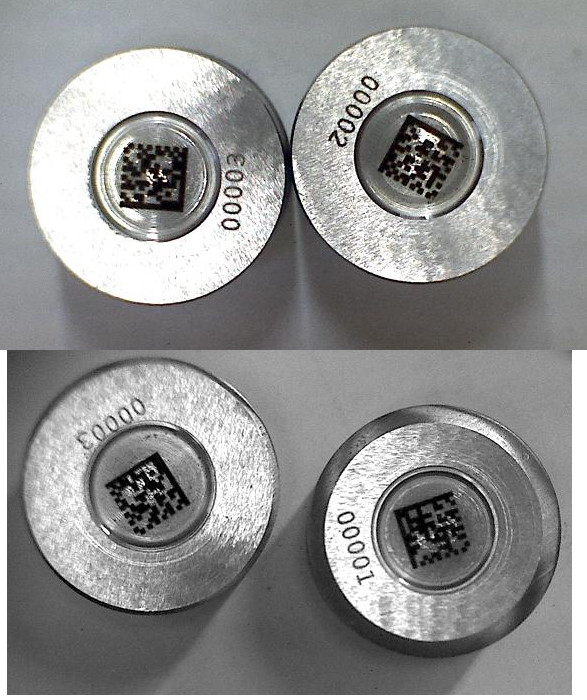

Supplement: Supplementary file 1 [file jimaging-07-00163-s001.zip › DMX3a.jpg]

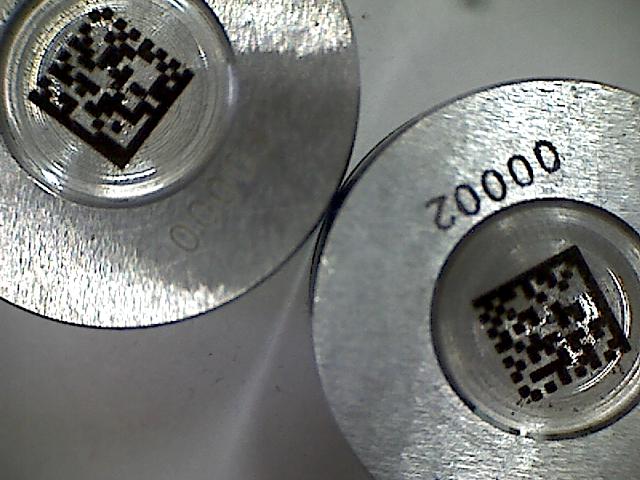

Supplement: Supplementary file 1 [file jimaging-07-00163-s001.zip › DMX3b_big.jpg]

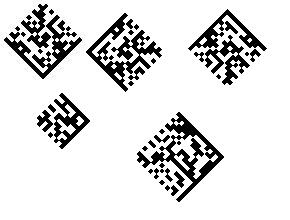

Supplement: Supplementary file 1 [file jimaging-07-00163-s001.zip › DMX1b.jpg]

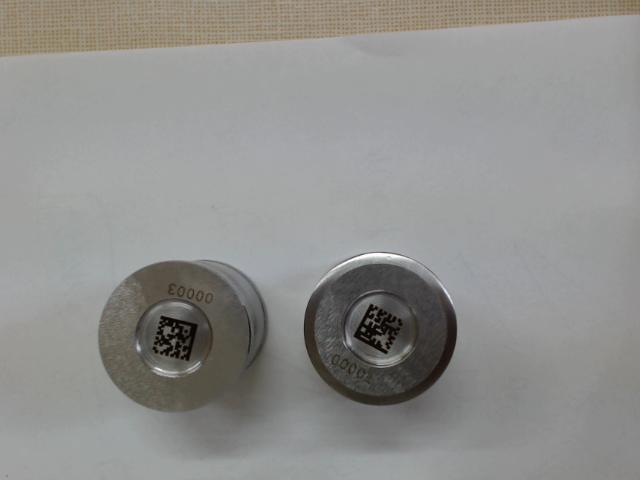

Supplement: Supplementary file 1 [file jimaging-07-00163-s001.zip › DMX2f_small.jpg]

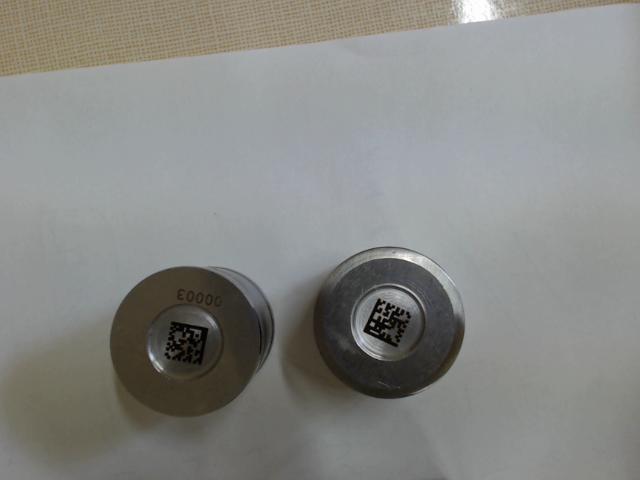

Supplement: Supplementary file 1 [file jimaging-07-00163-s001.zip › DMX2e_small.jpg]

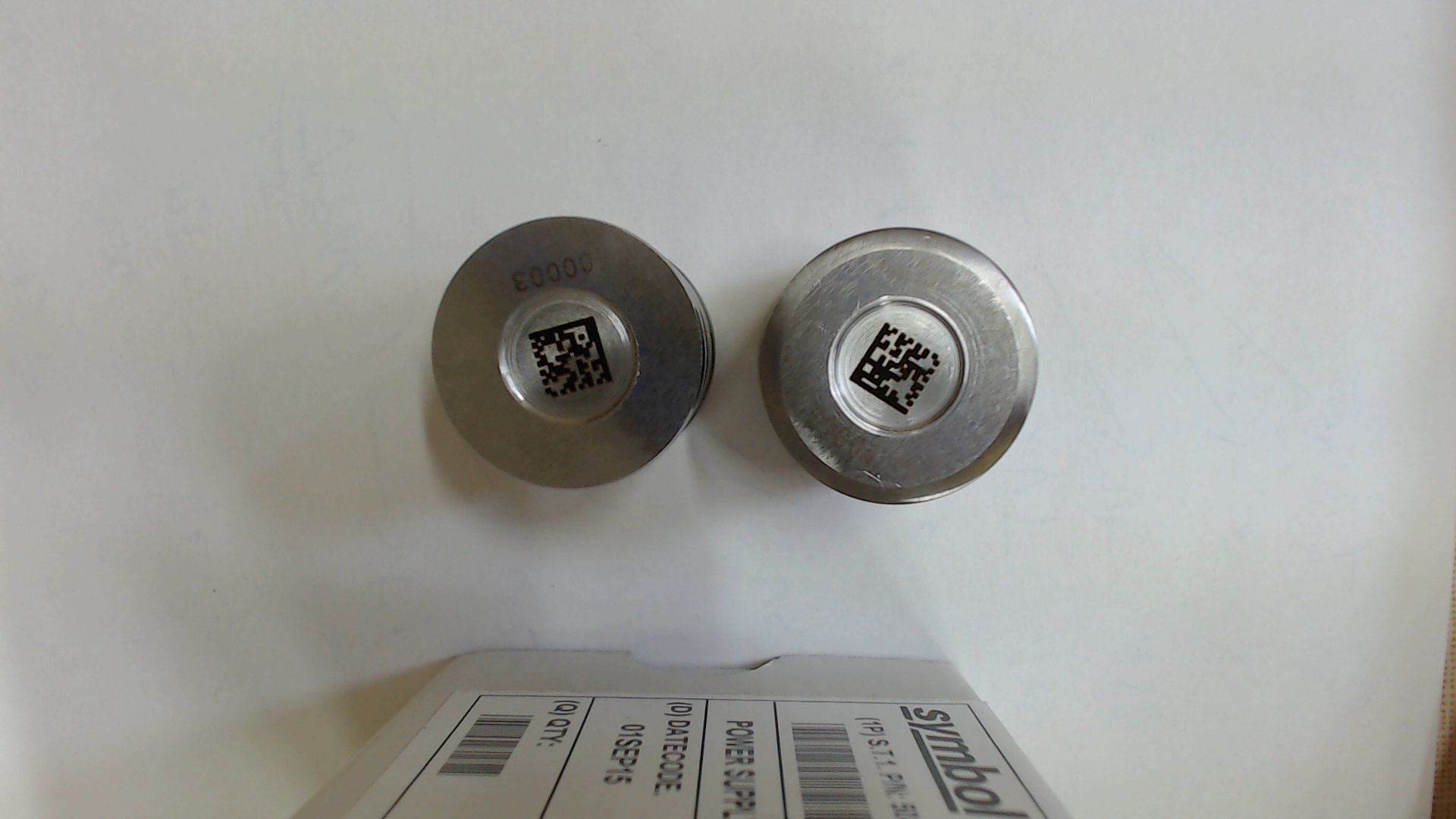

Supplement: Supplementary file 1 [file jimaging-07-00163-s001.zip › DMX2d_big.jpg]

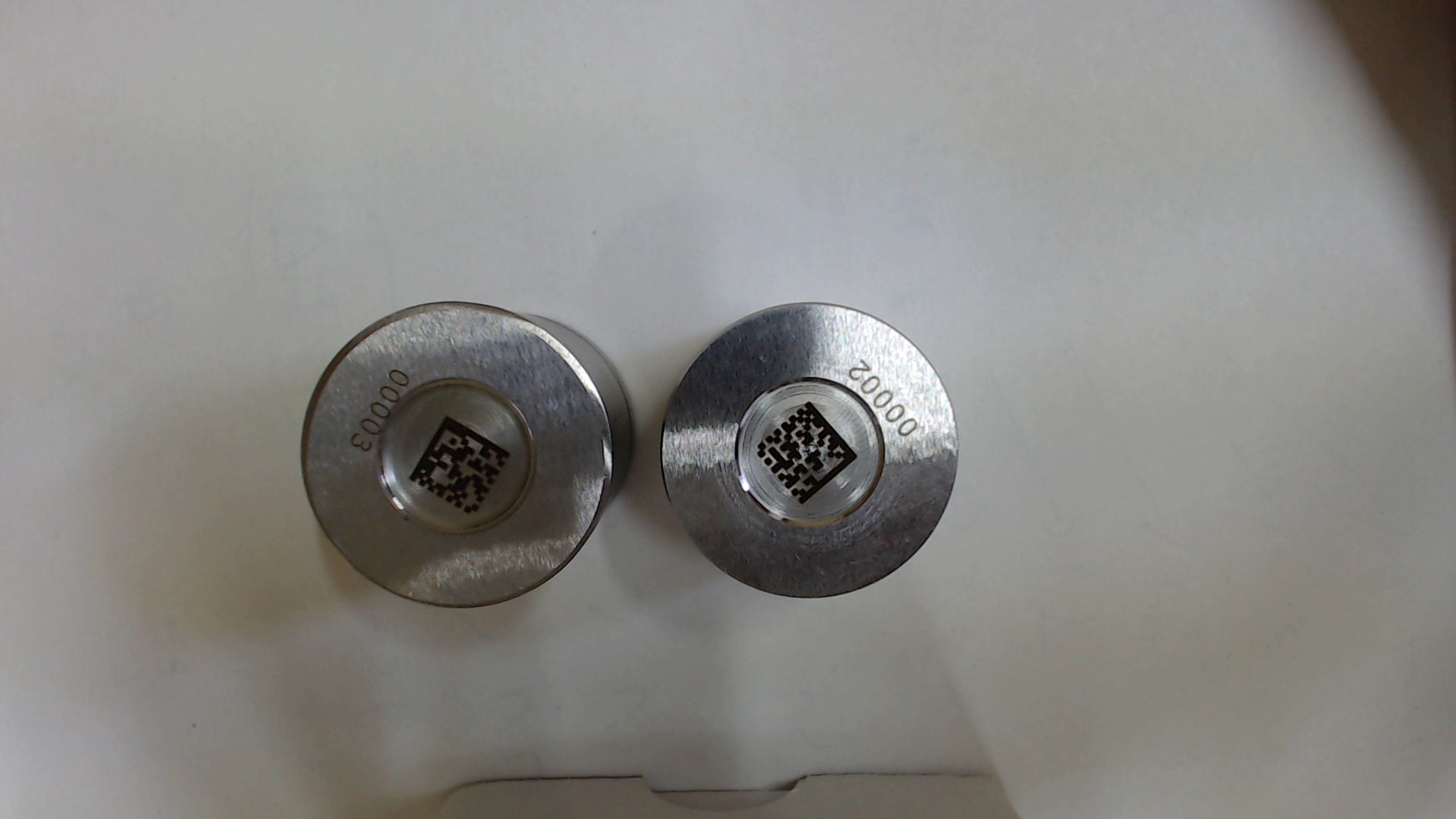

Supplement: Supplementary file 1 [file jimaging-07-00163-s001.zip › DMX2c_big.jpg]

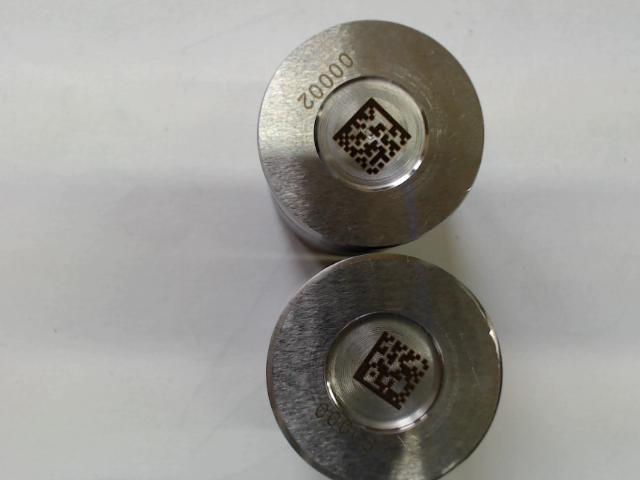

Supplement: Supplementary file 1 [file jimaging-07-00163-s001.zip › DMX2b.jpg]

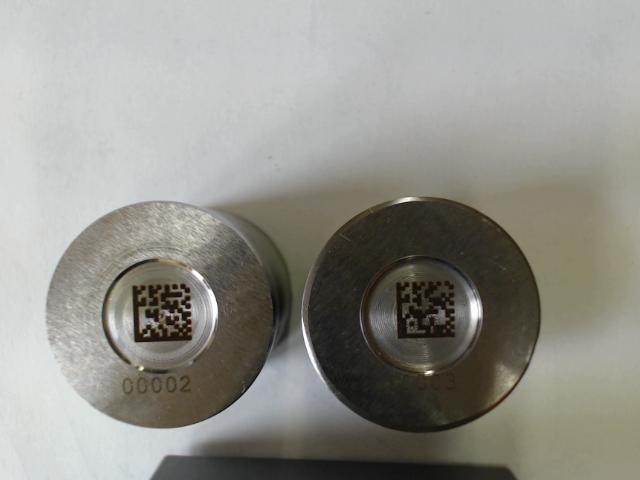

Supplement: Supplementary file 1 [file jimaging-07-00163-s001.zip › DMX2a.jpg]
